# LMNB2-mediated high PD-L1 transcription triggers the immune escape of hepatocellular carcinoma

**DOI:** 10.1038/s41420-025-02540-7

**Published:** 2025-06-07

**Authors:** Yuxuan Li, Jie Zhu, Fengguang Zhai, Yidong Ge, Ziqing Zhan, Shuyan Wang, Lili Kong, Jianan Zhao, Lecheng Hu, Siyuan Wang, Jiaxin Shi, Jianing Mao, Zongdong Yu, Haoyun Wang, Jiabei Jin, Mengxiang Zhao, Hong Li, Xiaofeng Jin

**Affiliations:** 1 Department of Hepatobiliary and Pancreatic Surgery, Ningbo Medical Center of LiHuiLi Hospital, Ningbo University, 315040 Ningbo, Zhejiang China; 2 Department of Biochemistry and Molecular Biology, Health Science Center, Ningbo University, 315211 Ningbo, Zhejiang China; 3 Department of Histopathology, Ningbo Clinical Pathology Diagnosis Center, 315040 Ningbo, Zhejiang China; 4 Department of Neurosurgery, Shangrao People’s Hospital, 334099 Shangrao, Jiangxi China; 5 Department of Stomatology, The First Affiliated Hospital of Ningbo University, Ningbo University, 315010 Ningbo, Zhejiang China

**Keywords:** Post-translational modifications, Immune evasion

## Abstract

While immune checkpoint inhibitors targeting programmed cell death-ligand 1 (PD-L1) demonstrate clinical efficacy in hepatocellular carcinoma (HCC), tumor cells frequently evade immune surveillance through PD-L1 overexpression, a phenomenon whose regulatory mechanisms remain poorly understood. Through integrated analysis of single-cell transcription sequence data, we identified aberrant upregulation of Lamin B2 (LMNB2) specifically in immunotherapy-sensitive HCC patients. Functional characterization revealed that LMNB2 acts as a transcriptional regulator of PD-L1, potentiating immune escape mechanisms in HCC cells during co-culture with Jurkat cells. Notably, we discovered that speckle-type POZ protein (SPOP) directly interacts with LMNB2 to mediate its ubiquitination and proteasomal degradation, thereby maintaining physiological PD-L1 expression levels. Clinically relevant *SPOP* mutations or reduced SPOP expression impaired this regulatory mechanism, leading to LMNB2 accumulation and subsequent PD-L1 hyperactivation. Importantly, combinatorial targeting of LMNB2 with Atezolizumab (PD-L1 inhibitor) displayed a synergistic effect on suppressing tumor progression both in vitro and in vivo, particularly in HCC models with *SPOP* mutations or LMNB2 overexpression. These findings unveil a novel ubiquitination-dependent regulatory axis in HCC immune evasion and propose targeted co-inhibition strategies to overcome HCC immunotherapy resistance.

## Introduction

Primary liver cancers are predominantly composed of hepatocellular carcinoma (HCC), which accounts for 75–85% of cases, and intrahepatic cholangiocarcinoma (ICC), which makes up 10–15% of cases [1]. In the early to middle stages, HCC patients can be treated radically through surgical resection, liver transplantation, radiofrequency ablation, or transcatheter chemoembolization [2]. However, most patients present with advanced cancer at diagnosis due to the late onset of symptoms, limiting them to systemic treatments [2]. In recent years, there have been significant advances in immune checkpoint blockade (ICB) therapy. Notably, immune checkpoint inhibitors, such as anti-programmed cell death-ligand-1 (PD-L1) antibodies, have demonstrated remarkable clinical efficacy in HCC treatment [3].

Although immunotherapy has achieved significant clinical efficacy in a subset of patients with HCC, the majority of patients do not respond to PD-1/PD-L1-targeted immunotherapy [4]. Specifically, tumor cells evade immune recognition by downregulating or losing tumor antigens and reducing major histocompatibility complex (MHC) expression [5, 6]. They overexpress immune checkpoint molecules like PD-L1 and Cytotoxic T-lymphocyte protein 4(CTLA4) to suppress T-cell function while recruiting immunosuppressive cells such as regulatory T cells (Tregs) and myeloid-derived suppressor cells (MDSCs) to create an immunosuppressive microenvironment through inhibitory cytokines [7, 8]. Besides, metabolic competition (e.g., glucose depletion, adenosine production, and lactate accumulation) and hypoxia further impair immune cell activity [9–11]. Immune checkpoint inhibitors targeting PD-L1/PD-1 counteract these mechanisms to restore antitumor immunity. However, the specific genetic alterations that drive aberrant overexpression of PD-L1 in cancer cells remain poorly understood.

Previous studies have demonstrated that PD-L1 expression is rigorously regulated through multiple mechanisms, including transcriptional, post-transcriptional, and post-translational levels [12, 13]. A variety of transcription factors play pivotal roles in inducing PD-L1 expression, such as interferon regulatory factor-1 (IRF1) [14], signal transducer and activator of transcription 1 (STAT1) [15], TEA domain family member 2 (TEAD) [16], and c-Myc [17]. A wealth of studies has confirmed that genetic or pharmacological inhibition of c-Myc can enhance anticancer immunity across different cancer types [18–20]. Similarly, inhibiting IRF1 has proven to boost immune surveillance in endometrial cancer [4] and non-small cell lung cancer [21]. Given these findings, it is evident that modulating the transcriptional regulation of PD-L1 holds great promise for uncovering novel therapeutic strategies. This could potentially amplify the efficacy of ICB therapies, leading to improved clinical outcomes in cancer patients by reinvigorating antitumor immune responses within the tumor microenvironment.

Cullin-RING E3 ligases (CRLs), which mediate the ubiquitination and degradation of a wide array of cellular regulators involved in numerous physiological and pathological processes, have a core structure comprising a cullin scaffold protein, a RING finger protein, and an adaptor that recruits specific substrate recognition subunits [22]. The speckle-type pox virus and zinc finger (POZ) protein (SPOP), as a substrate-binding adaptor of the Cullin 3-RING E3 ubiquitin ligase complex (CRL3), specifically identifies a short motif termed the SPOP-binding consensus (SBC) in its substrates via its N-terminal MATH domain [23]. The BTB and BACK domains of SPOP facilitate dimerization, interaction with CUL3, and self-oligomerization [24]. Of particular note, SPOP exerts tumor-suppressive effects by inducing the ubiquitination and degradation of oncoproteins, including c-Myc [25], androgen receptor (AR) [26], and estrogen receptor-α (ERα) [27]. In addition to its degradative functions, SPOP also performs tumor-suppressive roles by regulating the non-degradative ubiquitination of certain substrates, such as inverted formin 2 (INF2) [28] and myeloid differentiation primary response protein 88 (MyD88) [29]. However, mutations and pathologically low expression of SPOP are prevalent in various cancers. For instance, exon sequencing has revealed *SPOP* mutations in up to 13% of prostate cancers, where these mutations rank first among all altered genes and compromise substrate-binding capacity and oligomerization with SPOP [30]. This disruption leads to the dominant-negative inactivation of the entire CRL3-SPOP complex [31]. Moreover, SPOP expression is significantly downregulated in liver cancer [32] and colorectal cancer [33].

In mammals, the four principal lamin isoforms include Lamin A/C (LMNA/C), Lamin B1 (LMNB1), and Lamin B2 (LMNB2) [34]. B-type lamins (LMNB1 and LMNB2), are predominantly localized at the nuclear periphery, adjacent to the inner nuclear membrane. In contrast, A-type lamins, such as Lamin A/C, not only associate with the nuclear membrane but also extend into the nucleoplasm [34]. LMNB2 is implicated in diverse nuclear functions, including DNA replication and stability, transcriptional regulation, chromatin organization, and maintenance of nuclear stiffness [34]. Recent studies have suggested that downregulating LMNB1/2 expression may lead to a significant increase in LMNA mobility within the nucleoplasm [35]. This increased mobility might be crucial for compensating for LMNB1/2 deficiency to maintain chromatin organization and transcriptional activity. However, the underlying mechanism between lamin proteins and the transcription process remains to be elucidated. In this study, we identified that LMNB2 is markedly upregulated in HCC, is negatively correlated with immune infiltration, and is associated with poor prognosis in HCC patients. Mechanistically, we determined that LMNB2 serves as a substrate for the CRL3–SPOP complex, which mediates its degradation and ubiquitination. However, HCC-associated mutations or pathologically low expression of SPOP lead to aberrant stabilization of LMNB2, which in turn, directly induces PD-L1 transcription, thereby promoting tumor immune evasion of HCC. Furthermore, we identified LMNB2 as a potential therapeutic target. Notably, in both in vivo and in vitro models, the efficacy of ICB therapy was significantly enhanced in HCC with high LMNB2 expression.

## Result

### LMNB2 is significantly elevated in HCC, negatively related to immune infiltration

**Fig. 1:**
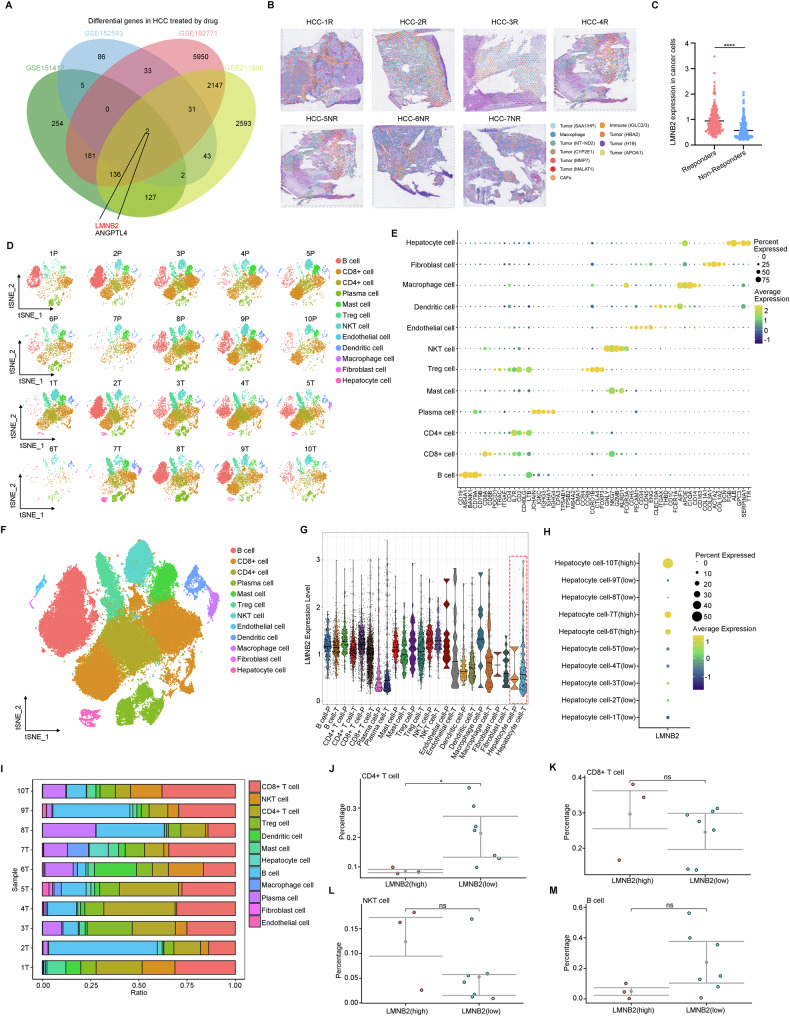
LMNB2 is significantly elevated in HCC, negatively related to immune infiltration. **A** Intersection of upregulated DEGs in drug-resistant HCC samples (GSE211850, GSE182593, GSE151412, and GSE192771). **B** Annotation and clustering for ST profiling. **C** Analysis expression of LMNB2 in HCC cells between patient responders and patient non-responders. **D**–**F** Debatching effect, clustering, and annotation for sc-RNA seq of 10 HCC samples (T) and 10 normal liver samples (P) (GSE235057). **G** Expression of LMNB2 in hepatocyte cells in sc-RNA seq of total10 HCC samples. **H** Expression of LMNB2 in hepatocyte cells in sc-RNA seq of separate 10 HCC samples. **I** Proportion of cell types in scRNA-seq of 10 HCC samples. **J** Proportion of CD4+ T cells in the 10 HCC samples grouped with high/low LMNB2 expression of hepatocyte cells. **K** Proportion of CD8+ T cells in the 10 HCC samples grouped with high/low LMNB2 expression of hepatocyte cells. **L** Proportion of NKT cells in the 10 HCC samples grouped with high/low LMNB2 expression of hepatocyte cells. **M** Proportion of B cells in the 10 HCC samples grouped with high/low LMNB2 expression of hepatocyte cells. Data are shown as mean ± SD (*n* ≥ 3). *p < 0.05, ns *p* ≥ 0.05.

To explore the genetic factors involved in drug resistance in liver cancer, we conducted a comprehensive analysis of differentially expressed genes (DEGs) across multiple liver cancer GEO datasets (GSE211850, GSE182593, GSE151412, and GSE192771), with a focus on drug resistance to Sorafenib and Renvastinib. Our analysis revealed that LMNB2 and Angiopoietin-related protein 4 (ANGPTL4) showed significant changes (Fig. 1A). Further investigation of spatial transcriptomics (ST) profiling (GSE238264) in immune-responsive and non-responsive HCC patients uncovered that LMNB2 expression was elevated in responders compared to non-responders (Fig. 1B and C). Furthermore, we analyzed the proportion of immune cells in the LMNB2 high/low expression groups using sc-RNA seq data. First, the data of ten liver cancer samples from the GEO database (GSE235057) were integrated, and the batch effect was removed (Fig. 1D). The total sample was then reduced, clustered, and annotated, and individual cell clusters were labeled (Fig. 1E and F). Upon examining LMNB2 distribution across various cell types, we observed higher LMNB2 expression in hepatocytes of HCC tissue than in those of normal liver tissue (Fig. 1G). The expression of LMNB2 in hepatocyte cells of 10 samples of HCC tissues was compared, and 6-/7-/10-HCC samples were regarded as the high expression LMNB2 groups, while 1-/2-/3-/4-/5-/8-/9-HCC samples was regarded as the low expression LMNB2 groups (Fig. 1H). Notably, in the high-LMNB2-expressing hepatocyte group, we found a reduced proportion of CD4^+^ T cells (Fig. 1I–M).

Moreover, our analysis uncovered that LMNB2 is significantly upregulated in liver cancer and exhibits a strong correlation with unfavorable prognosis. Data retrieved from the TCGA and GEO databases (GSE76427 and GSE45627) revealed markedly higher LMNB2 levels in liver cancer tissues than in normal liver tissues (Fig. S1A–C). These findings were further validated through immunohistochemical staining and WB analysis, which confirmed the aberrantly high expression of LMNB2 in liver cancer (Fig. S1D–J). Importantly, patients with elevated LMNB2 expression demonstrated poor prognosis (Fig. S1K–N). Additionally, key clinical characteristics, including pathological and histological grade, AFP levels, and tumor size, showed a close association with LMNB2 expression (Fig. S1O, P and Table 1).

**Table 1:** Summary of clinical features for 60 matched HCC sample pairs.

Clinical features	Number	LMNB2			*χ*^2^	*p*-value
		Negative	Low positive	High positive		
*n*	60	9	28	23		
*Gender*					1.7313	0.4208
Female	9	1	6	2		
Male	51	8	22	21		
*Age*					4.1197	0.1275
>50	55	9	27	19		
≤50	5	0	1	4		
*Tumor number*					1.636	0.4413
1	59	9	28	22		
2	1	0	0	1		
*Tumor size (cm)*					7.6032	***0.02234***
>5	22	0	10	12		
≤5	38	9	18	11		
*Grade stage*					4.5122	0.1048
G1	14	1	10	3		
G2	46	8	18	20		
*Metastasis*					4.9464	0.2928
M0	39	7	20	12		
M1	19	9	8	2		
M2	2	2	0	0		
*AFP*					2.1859	0.7016
−	28	4	12	12		
+	7	0	4	3		
Unknown	25	5	12	8		
*β-Catenin*					5.9596	0.0508
+	20	2	6	12		
Unknown	40	7	22	11		
*CD10*					6.7579	0.1492
−	17	0	8	9		
+	17	2	9	6		
Unknown	26	7	11	8		
*CD34*					4.0217	0.4031
−	3	0	1	2		
+	33	3	17	13		
Unknown	24	6	10	8		
*CK7*					1.8997	0.7542
−	18	1	9	8		
+	15	3	7	5		
Unknown	27	5	12	10		
*GS*					3.8926	0.4207
−	11	1	6	4		
+	31	3	14	14		
Unknown	18	5	8	5		
*KI67*					5.7311	0.454
+	7	1	4	2		
++	27	3	13	11		
+++	8	0	3	5		
Unknown	18	5	8	5		
*CK19*					4.7067	0.3187
−	36	4	16	16		
+	6	0	4	2		
Unknown	18	5	8	5		
*GPC3*					6.2584	0.1807
−	7	2	2	3		
+	35	15	18	2		
Unknown	18	5	8	5		
*P53*					1.9057	0.7531
−	9	0	5	4		
+	24	4	11	9		
Unknown	27	5	12	10		
*Arginase 1*					4.1153	0.3906
−	5	0	3	2		
+	37	4	17	16		
Unknown	18	5	8	5		

Bold italic values indicate statistical significance.

### LMNB2 can promote tumor growth by inducing T-cell apoptosis

**Fig. 2:**
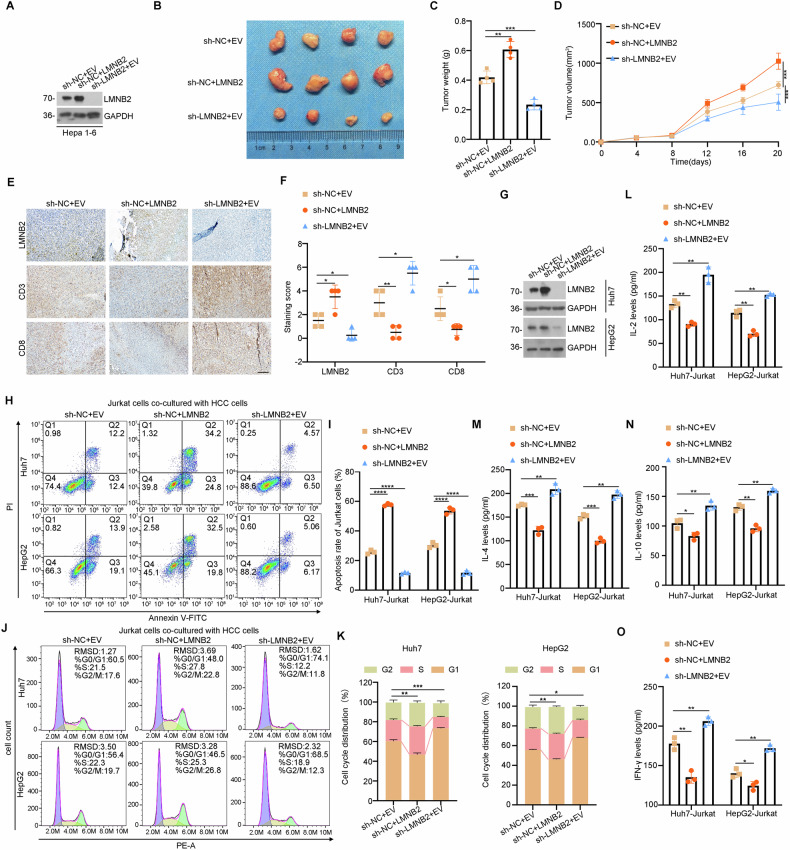
LMNB2 can promote tumor growth by inducing T-cell apoptosis. **A** WB verified the overexpression and knockdown efficiency of LMNB2 in Hepa1-6 cells. **B** Schematic representation of xenograft tumors with sh-NC + EV, sh-NC + LMNB2, and sh-LMNB2 + EV. **C** Weight of xenograft tumors of sh-NC + EV, sh-NC + LMNB2, and sh-LMNB2 + EV. **D** Volume of xenograft tumors of sh-NC + EV, sh-NC + LMNB2, and sh-LMNB2 + EV. **E** IHC staining of LMNB2, CD3, and CD8 in sh-NC + EV, sh-NC + LMNB2, and sh-LMNB2 + EV in xenograft tumors. Scale bar, 200 μm. **F** IHC staining scores for LMNB2, CD3, and CD8 in sh-NC + EV, sh-NC + LMNB2, and sh-LMNB2 + EV in xenograft tumors. **G** WB verified the overexpression and knockdown efficiency of LMNB2 in Huh7 and HepG2 cell lines. **H** Apoptosis of Jurkat cells co-cultured with Huh7/HepG2 cells treated with sh-NC + EV, sh-NC + LMNB2, or sh-LMNB2 + EV was detected by flow cytometry. **I** Statistical analysis of apoptotic levels in Jurkat cells (**H**). **J** The cell cycle of Jurkat cells co-cultured with Huh7/HepG2 cells treated with sh-NC + EV, sh-NC + LMNB2, and sh-LMNB2 + EV was analyzed by flow cytometry. **K** Cell cycle statistics of Jurkat cells (**J**). **L**–**O** The levels of IL-2, IL-4, IL-10, and IFN-γ produced by Jurkat cells were detected using ELISA. Data are shown as mean ± SD (*n* ≥ 3). *p < 0.05, **p < 0.01, ***p < 0.001, ****p < 0.0001.

To explore the impact of LMNB2 on immune infiltration, we manipulated LMNB2 expression in mouse hepatoma Hepa1-6 cells, creating overexpression and knockdown groups (Fig. 2A). These cells were then injected into C57BL/6J mice to establish subcutaneous tumor models. Analysis of tumor weight and volume indicated that LMNB2 significantly drove liver cancer tumor progression (Fig. 2B–D). Assessment of T cell activation biomarkers CD3 and CD8 [36] revealed reduced immune infiltration in the LMNB2-overexpressing group (Fig. 2E and F). In co-culture experiments with Jurkat cells and liver cancer cell lines (Huh7 and HepG2) with varying LMNB2 expression levels (Fig. 2G), elevated LMNB2 was found to substantially induce T cell apoptosis (Fig. 2H and I) and cell cycle arrest (Fig. 2J and K). Additionally, co-culture supernatant analysis showed that high LMNB2 expression markedly diminished the secretion of inflammatory cytokines interleukin-2/4/10 (IL-2/4/10) and interferon-γ (IFN-γ) (Fig. 2L–O). Furthermore, functional phenotype assays demonstrated that increased LMNB2 levels facilitated HCC occurrence and progression in vitro (Fig. S2). Collectively, these data indicate that elevated LMNB2 levels promote tumor growth both in vitro and in vivo.

### LMNB2 is a bona fide transcription factor of PD-L1

**Fig. 3:**
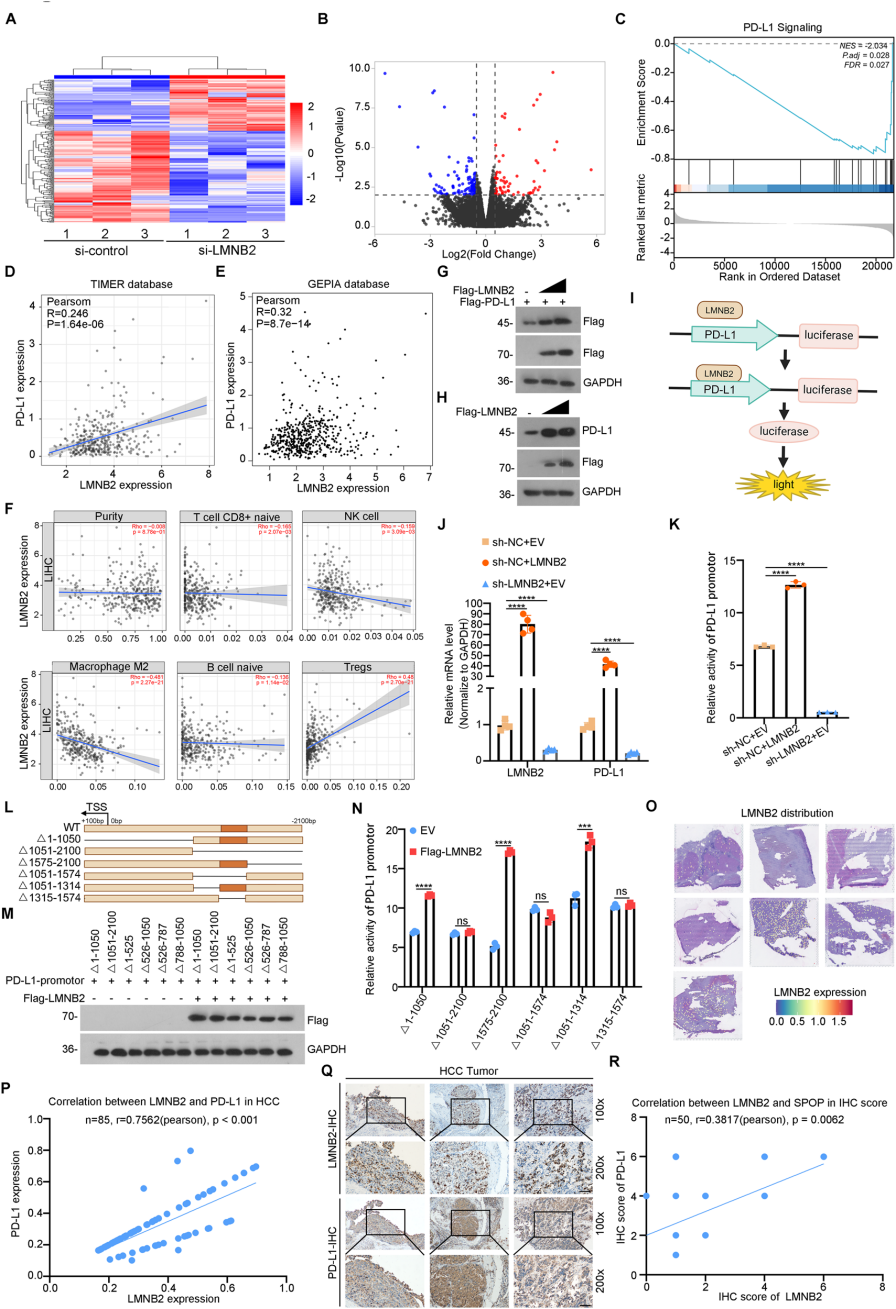
LMNB2 is a bona fide transcription factor of PD-L1. **A** RNA-seq analysis of control and LMNB2 knockdown Huh7 cells. **B** Volcano map of DEGs in control and LMNB2 knockdown Huh7 cells. **C** GSEA analysis of PD-L1 signaling in control and LMNB2 knockdown Huh7 cells. **D** Correlation analysis between LMNB2 and PD-L1 expression in the TIMER database. **E** Correlation analysis between LMNB2 and PD-L1 expression in the GEPIA database. **F** Correlation analysis between LMNB2 and immune cell infiltration in HCC using the TIMER database. **G** LMNB2 promotes the protein expression of exogenous PD-L1 in HepG2 cells. **H** LMNB2 promotes the protein expression of endogenous PD-L1 in HepG2 cells. **I** Model diagram of LMNB2 binding to the PD-L1 promoter to promote its transcription. **J** LMNB2 promotes the mRNA expression of PD-L1. **K** LMNB2 promotes the activity of PD-L1. **L** Model diagram of the six truncated PD-L1 promoters. **M** WB verified the overexpression of LMNB2 in 293T cells for dual-luciferase reporter analysis. **N** Statistical of the LMNB2 effect on the activity of six truncated PD-L1 promoters (**O**). Distribution of LMNB2 in ST-RNA-seq of HCC samples. **P** Correlation analysis between LMNB2 and PD-L1 in ST-RNA-seq in HCC samples. **Q** IHC staining of LMNB2 and PD-L1 in HCC samples. Scale bar, 200 μm. **R** Correlation analysis between LMNB2 and SPOP IHC scores. Data are shown as mean ± SD (*n* ≥ 3). ***p < 0.001, ****p < 0.0001.

Previous studies have demonstrated that LMNB2 can enhance the transcriptional activity of specific genes [37]. To delineate the pathways through which LMNB2 impacts liver cancer, we performed RNA-seq on Huh7 cells with LMNB2 knockdown and corresponding control cells. Pronounced differences in gene expression were observed between the two groups (Fig. 3A and B). Notably, the PD-L1 signaling pathway was markedly downregulated in liver cancer cells following LMNB2 knockdown (Fig. 3C). This suggested that LMNB2-mediated immune evasion in liver cancer might be linked to PD-L1 expression. Analysis of the TIMER and GEPIA databases further confirmed a positive correlation between LMNB2 and PD-L1 (Fig. 3D and E). Moreover, TIMER database analysis of immune infiltration in liver cancer revealed that high LMNB2 expression was frequently associated with reduced immune cell infiltration in the tumor microenvironment (Fig. 3F). We also found that exogenous PD-L1 protein levels increased with elevated LMNB2 expression, paralleling endogenous PD-L1 protein levels in HepG2 cells (Fig. 3G and H). Dual-luciferase reporter assays showed that LMNB2, acting as a transcription factor, upregulated PD-L1 mRNA expression by binding to its promoter region (Fig. 3I–K). To identify the specific binding sites between LMNB2 and PD-L1 promoter, we truncated the PD-L1 promoter into regions P1(△1–1050), P2(△1051–2100), P3(△1575–2100), P4(△1051–1574), P5(△1051–1314), and P6(△1315–1574) (Fig. 3L). We determined that the P6 region of the PD-L1 promoter served as the binding site for LMNB2 (Fig. 3M and N). Moreover, ST profiling and IHC analysis validated the positive correlation between LMNB2 and PD-L1 in liver cancer (Fig. 3O–R).

### SPOP recognizes LMNB2-417“ATSSS”-421 for lysosome-mediated degradation through K29-linkage ubiquitination

**Fig. 4:**
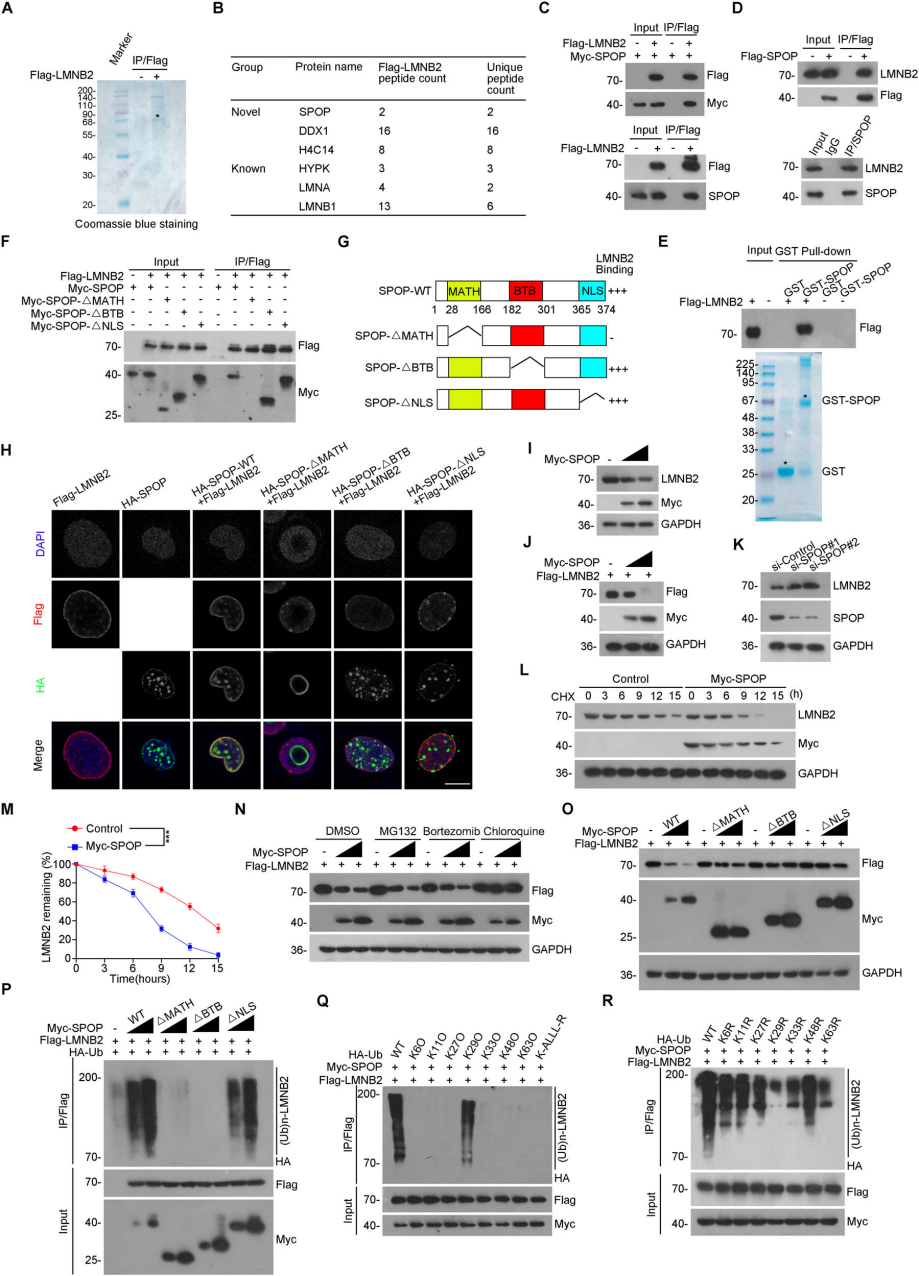
SPOP promotes LMNB2 lysosome-mediated degradation through K29-linkage ubiquitination. **A** Coomassie blue staining for the enrichment of LMNB2. **B** Mass spectrometry of related proteins in LMNB2 enrichment. **C** and **D** Interaction between LMNB2 and SPOP. **E** Interaction between LMNB2 and SPOP in vitro. **F** Interaction between LMNB2 and SPOP-WT, SPOP-ΔMATH, SPOP-ΔBTB, and SPOP-ΔNLS. **G** Mode diagram of binding ability between LMNB2 and SPOP-WT, SPOP-ΔMATH, SPOP-ΔBTB, or SPOP-ΔNLS. **H** Co-localization of LMNB2, SPOP-WT, SPOP-ΔMATH, SPOP-ΔBTB, and SPOP-ΔNLS. Scale bar, 50 μm. **I**–**K** SPOP promotes the protein expression of LMNB2. **L** and **M** Effect of SPOP on the half-life of LMNB2. **N** Identifying degradation pathway of LMNB2 induced by SPOP. **O** Effects of SPOP-WT, SPOP-ΔMATH, SPOP-ΔBTB, and SPOP-ΔNLS on LMNB2 degradation. **P** Effects of SPOP-WT, SPOP-ΔMATH, SPOP-ΔBTB, and SPOP-ΔNLS on LMNB2 ubiquitination. **Q** and **R** Identification of ubiquitination linkage of LMNB2 induced by SPOP. Data are shown as mean ± SD (*n* ≥ 3). ***p < 0.001.

The underlying mechanism of LMNB2 overexpression in liver cancer remains unclear. To figure out this, tandem affinity purification coupled with mass spectrometry (TAP-MS) was employed to identify potential LMNB2-interacting proteins. Among the candidates, SPOP, which has been implicated in immune evasion in liver and endometrial cancers [4, 36], was prioritized (Fig. 4A and B). The interaction between LMNB2 and SPOP was validated via Co-IP assays at both exogenous and endogenous levels (Fig. 4C and D). Additionally, GST pull-down assays confirmed the in vitro interaction between PD-L1 and LMNB2 (Fig. 4E).

SPOP comprises an N-terminal MATH domain for substrate recognition, a C-terminal BTB domain that binds CUL3 to form a ubiquitin ligase complex, and an NLS domain for nuclear localization [23]. Co-IP assays with SPOP domain truncations revealed that the MATH domain was essential for the interaction with LMNB2 (Fig. 4F and G). IF assays further demonstrated that SPOP-Wild Type (WT) and LMNB2 co-localized in the nucleus, a pattern not observed with SPOP-ΔMATH (Fig. 4H).

To explore whether SPOP regulates LMNB2 protein levels, we conducted gradient transfections of Myc-SPOP in HEK-293T cells. The results revealed a corresponding decrease in both endogenous and exogenous LMNB2 expression (Fig. 4I and J). Consistently, LMNB2 expression increased when SPOP levels were reduced (Fig. 4K). Additionally, SPOP overexpression shortened the half-life of LMNB2, confirming that SPOP regulates LMNB2 protein levels (Fig. 4L and M). Further analysis showed this regulation occurs through lysosome-mediated degradation (Fig. 4N). LMNB2 ubiquitination and degradation were mediated by SPOP-WT but not by SPOP-ΔMATH or ΔBTB mutants. Interestingly, SPOP-ΔNLS also mediated LMNB2 ubiquitination and degradation, albeit less efficiently than SPOP-WT (Fig. 4O and P). Notably, SPOP-mediated LMNB2 ubiquitination mainly involved K29-linkage polyubiquitin chains, suggesting a potential role in lysosome-associated receptor protein recognition (Fig. 4Q and R).

To further elucidate the regulatory mechanism of LMNB2 by SPOP, we utilized the PhosphoSitePlus database (https://www.phosphosite.org/) to predict potential ubiquitination sites in LMNB2 and identified 23 candidate sites (Fig. S3A). TAP-MS analysis revealed seven specific Lys residues in LMNB2 as putative ubiquitination sites mediated by SPOP (Fig. S3B–J). Collectively, these results demonstrate that the MATH, BTB, and NLS domains of SPOP are essential for mediating K29-linkage polyubiquitination of LMNB2 and its subsequent lysosomal degradation.

Previous studies have reported that SPOP substrates contain one or more SBC motifs (θ-π-S–S/T-S/T; θ: nonpolar residues, π: polar residues) [24]. To identify the LMNB2 sequence responsible for SPOP interaction, we detected a potential LMNB2 fragment (amino acids 417-ATSSS-421), which is the minimal region required for SPOP binding and resembles known SPOP substrate motifs (Fig. S4A and B). To examine whether this potential motif is required for the SPOP-LMNB2 interaction, we generated an *LMNB2* mutant in which the motif sequence was deleted (LMNB2-ΔSBC), which completely abolished SPOP-mediated LMNB2 interaction, degradation, and ubiquitination (Fig. S4C–F). Consistently, LMNB2 half-life was significantly prolonged upon SBC motif deletion (Fig. S4G and H). GST pull-down assays confirmed that LMNB2-ΔSBC lost its ability to interact with SPOP (Fig. S4I). Additionally, amino acid substitution within the SBC motif reduced LMNB2-SPOP interaction and impaired SPOP-mediated LMNB2 degradation and ubiquitination (Fig. S4J–L). Notably, several pathological mutations at the LMNB2 SBC site have been reported in the COSMIC database (https://cancer.sanger.ac.uk/cosmic), implying their potential role in disease development. Overall, our findings demonstrate that the conserved SBC motif of LMNB2 serves as a degron recognized by SPOP, which is mediated via K29-linkage polyubiquitination and lysosomal degradation of LMNB2.

### Mutations and pathological low expression of SPOP induce immune escape of HCC cells in an LMNB2‑PD‑L1 axis-dependent manner

Our exploration into the COSMIC database revealed four missense mutations in the MATH domain of SPOP (M35L, S119N, F136L, D153Y) linked to HCC (Fig. S5A). We postulated that HCC-associated SPOP mutants may be defective in mediating LMNB2 turnover. As expected, LMNB2 binding ability of these *SPOP* mutants, especially M35L and S119N, was drastically reduced compared to SPOP-WT (Fig. S5B). Consequently, SPOP-mediated LMNB2 degradation and ubiquitination were significantly diminished (Fig. S5C and D). IF analysis showed that HCC-associated *SPOP* mutants failed to recruit LMNB2 into nuclear speckles (Fig. S5E). Consistent with prior studies [4], co-expression of SPOP-M35L and S119N mutants markedly reduced the interaction between SPOP-WT and LMNB2, suppressing SPOP-mediated LMNB2 degradation and ubiquitination (Fig. S5F–H). Furthermore, SPOP expression was lower in HCC tissues than in paired normal liver tissues derived from 14 pairs of fresh human tissues (Fig. S5I and J). Consistent with this, the IHC of 60 pairs of HCC and paired normal liver tissues showed that SPOP protein expression was downregulated in a large percentage of HCC tissues (Fig. S5K and L). Similarly, SPOP expression was found to be abnormally low in liver cancer cell lines compared to normal liver cells (Fig. S5M and N). Importantly, IHC staining analysis revealed a negative correlation between SPOP and LMNB2 expression in liver cancer tissues (Fig. S5O and P). Finally, we confirmed that SPOP-WT could reverse LMNB2’s carcinogenic effect in HCC, but SPOP-M35L failed (Fig. S6).

**Fig. 5:**
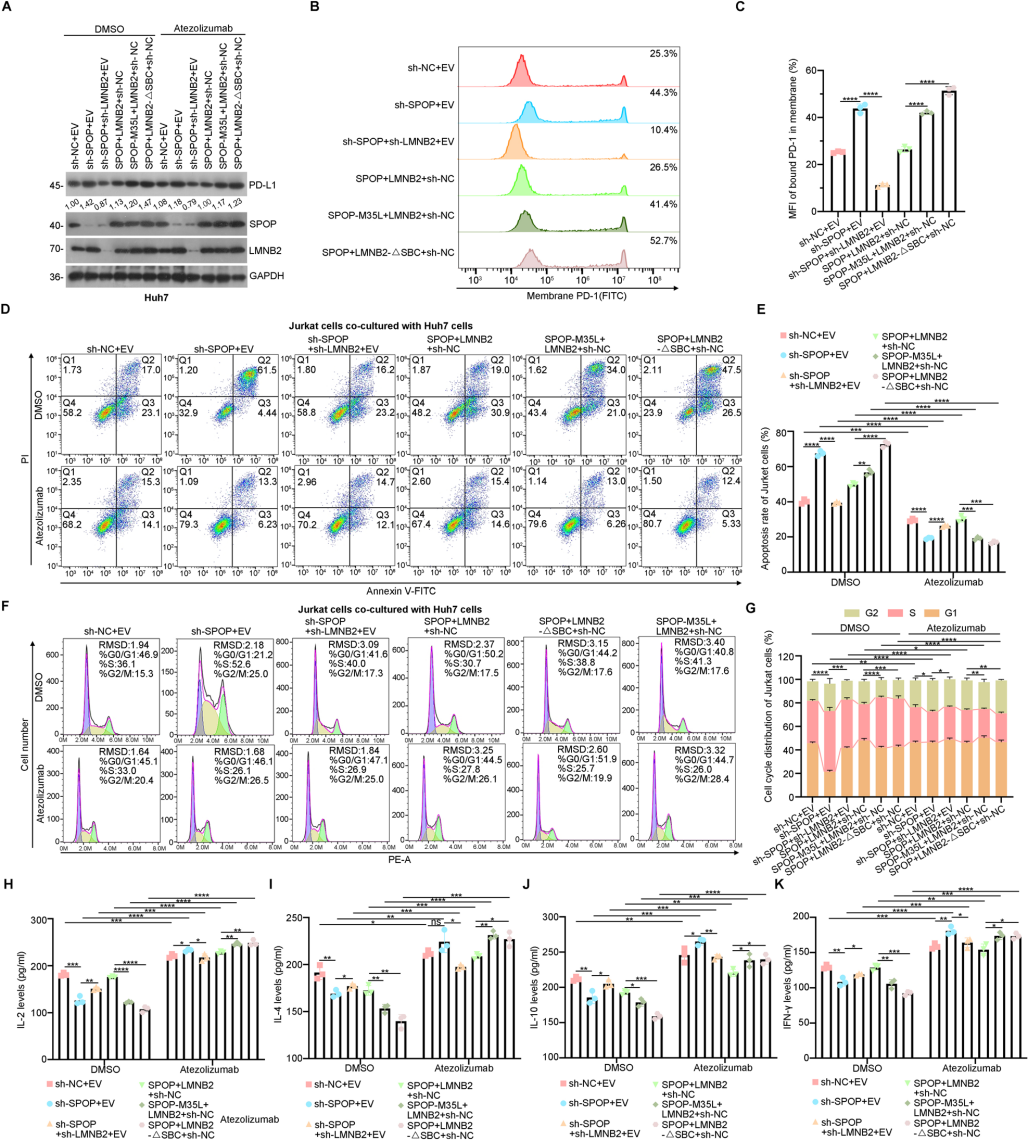
SPOP-induced immune surveillance depends on LMNB2‑PD‑L1 axis in Huh7 cells. **A** Huh7 cells achieving sh-NC + EV, sh-SPOP + EV, sh-SPOP+sh-LMNB2 + EV, SPOP + LMNB2 + sh-NC, SPOP + LMNB2-△SBC+sh-NC and SPOP-M35L + LMNB2 + sh-NC were co-cultured with Jurkat cells for 24 h after treatment with DMSO or Atezolizumab (10 ng/mL). WB of Huh7 cells in the HCC cell-Jurkat cell co-culture system for the detection of PD-L1 expression levels. All quantitation was normalized to the protein level of GAPDH in the sh-NC + EV group. **B** Huh7 cells achieving the above treatment were co-cultured with Jurkat cells for 24 h. Flow cytometry analysis of PD-1 binding on Huh7 cell surface. **C** Statistics of mean fluorescence intensity (MFI) for PD-1 in (**B**). **D** Huh7 cells achieving the above treatment were co-cultured with Jurkat cells for 24 h after treatment with DMSO or Atezolizumab (10 ng/ml). Apoptosis levels in Jurkat cells were detected by flow cytometry. **E** Statistical analysis of apoptotic levels in Jurkat cells (**D**). **F** Huh7 cells achieving the above treatment were co-cultured with Jurkat cells for 24 h after treatment with DMSO or Atezolizumab (10 ng/ml). The cell cycles of Jurkat cells were detected by flow cytometry. **G** Cell cycle statistics of Jurkat cells (**F**). **H**–**K** The levels of IL-2, IL-4, IL-10, and IFN-γ produced by Jurkat cells were detected using ELISA. Data are shown as mean ± SD (*n* ≥ 3). *p < 0.05, **p < 0.01, ***p < 0.001, ****p < 0.0001.

To investigate the role of the SPOP-LMNB2-PD-L1 axis in HCC tumor immunity, we used a co-culture model. Overexpressing LMNB2 in Huh7 cells increased PD-L1 expression compared to controls, while LMNB2 knockdown had the opposite effect. Notably, LMNB2 knockdown reversed the PD-L1 upregulation caused by SPOP depletion (Fig. 5A). Additionally, co-culture experiments showed that LMNB2 significantly increased PD-1 expression on Jurkat cells (Fig. 5B and C). Further analysis revealed that LMNB2 promoted apoptosis (Fig. 5D and E) and blocked G1/S cell cycle progression in Jurkat cells (Fig. 5F and G), with HCC-associated *SPOP* mutants and SBC-truncated LMNB2 exacerbating these effects. However, these effects were reversed by the PD-L1 inhibitor Atezolizumab, particularly in high-LMNB2-expressing groups. We also found that cytokine secretion (IL-2, IL-4, IL-10, IFN-γ) from Jurkat cells was significantly higher in the LMNB2/SPOP co-knockdown group than in the SPOP knockdown group alone (Fig. 5H–K), indicating that SPOP-induced immune surveillance partially depends on LMNB2. Atezolizumab effectively counteracted the effects of elevated LMNB2 on cytokine secretion. Similar results were observed in HepG2 cells (Fig. S7). Overall, our data demonstrate that SPOP-induced immune surveillance relies on the LMNB2–PD-L1 axis and that the detrimental effects of this axis can be reversed by Atezolizumab in HCC in vitro.

### Atezolizumab counteracts defective SPOP–LMNB2–PD-L1 axis-induced immune escape of HCC in vivo

**Fig. 6:**
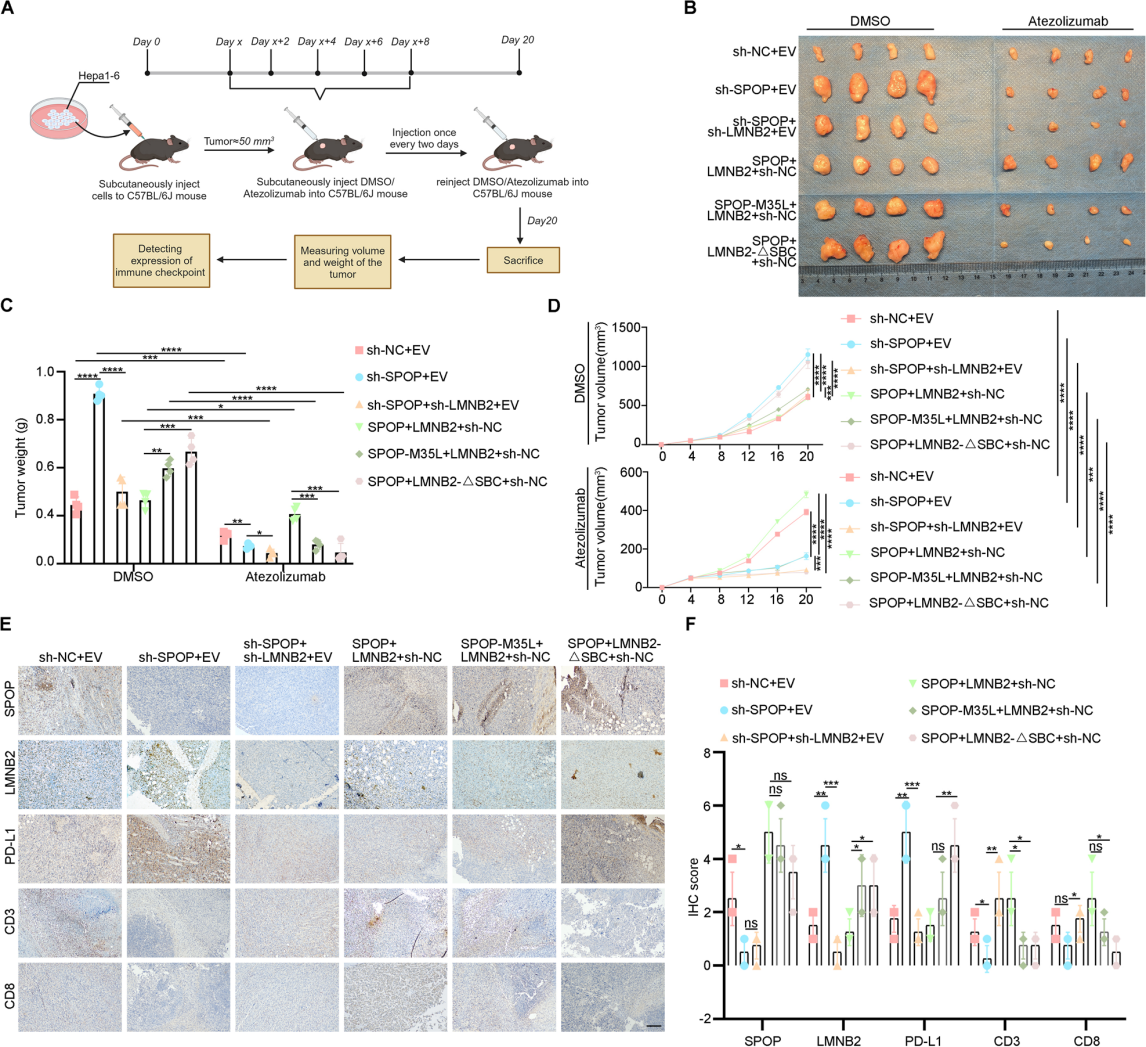
Atezolizumab counteracts defective SPOP-LMNB2-PD-L1 axis-induced immune escape of HCC in vivo*.* **A** Mode diagram of Atezolizumab injection in C57BL/6 J mice with a subcutaneous tumor. **B** Schematic representation of xenograft tumors of sh-NC + EV, sh-SPOP + EV, sh-SPOP + sh-LMNB2 + EV, SPOP + LMNB2 + sh-NC, SPOP + LMNB2-△SBC + sh-NC, and SPOP-M35L + LMNB2 + sh-NC groups in DMSO or Atezolizumab. **C** Weight of xenograft tumors in the above groups. **D** Volume of xenograft tumor in the above group. **E** IHC staining of SPOP, LMNB2, PD-L1, CD3, and CD8 in xenograft tumors. Scale bar, 200 μm. **F** Statistical analysis of the IHC staining scores of SPOP, LMNB2, PD-L1, CD3, and CD8 in xenograft tumors. Data are shown as mean ± SD (*n* ≥ 3). *p < 0.05, **p < 0.01, ***p < 0.001, ****p < 0.0001, ns *p* ≥ 0.05.

In addition, we further utilized animal models to elucidate the effect of Atezolizumab on SPOP-LMNB2-PD-L1 signal axis disruption in vivo. Hepa1-6 cells were injected subcutaneously into mice. When tumors reached ~50 mm³, Atezolizumab or DMSO (control) was administered every two days. Mice were sacrificed on day 20 (Fig. 6A). Results showed that differences in tumor weights among the sh-NC + EV, sh-SPOP + EV, and sh-SPOP+sh-LMNB2 + EV groups were significantly reduced with Atezolizumab treatment, indicating that elevated LMNB2 promotes tumor immune evasion (Fig. 6B–D). Similarly, Atezolizumab mitigated tumor weight differences between the SPOP + LMNB2 + sh-NC and SPOP-M35L + LMNB2 + sh-NC groups, suggesting that *SPOP* mutations in HCC promote immune evasion partly through LMNB2 (Fig. 6B–D). Besides IHC analysis of tumor tissues revealed that LMNB2 upregulated PD-L1 and downregulated immune markers CD3 and CD8 (Fig. 6E and F). Overall, these animal experiments demonstrate that LMNB2, which is highly expressed in HCC with *SPOP* mutations or low SPOP expression, promotes tumorigenesis. However, this effect is reversed by Atezolizumab, with greater efficacy in HCC with high LMNB2 expression. Thus, Atezolizumab effectively counteracts immune evasion mediated by SPOP–LMNB2–PD-L1 axis defects in HCC in vivo.

To delve into the immune role of Lmnb2 in the mouse HCC model, we modulated its expression in Hepa1-6 cells and conducted in vivo xenograft assays and sc-RNA seq analysis (Fig. S8A–C). Consistent with previous results, high Lmnb2 expression was linked to elevated Pd-l1 levels (Fig. S8D). We also analyzed the expression of Il-2, Il-4, Il-10, Ifn-γ, and Pd-1 on T cells in sh-Lmnb2 and OE-Lmnb2 groups. Ifn-γ and Pd-1 on T cells were markedly upregulated in the OE-Lmnb2 group, while Il-2, Il-4, and Il-10 levels showed no significant changes (Fig. S8E–I). Correlation analysis revealed a positive relationship between Lmnb2 and Pd-l1/Pd-1 (Fig. S8J). Given that IFN-γ can induce PD-L1 expression and promote tumor immune evasion [38, 39], LMNB2 may not only directly upregulate PD-L1/PD1 but also enhance immune evasion in HCC cells by boosting IFN-γ expression on T cells. Moreover, cell communication analysis indicated that HCC cells with high Lmnb2 expression exhibited significantly enhanced Pd-l1 signaling communication with T cells (Fig. S8K–N).

## Discussion

The expression levels of PD-L1 in tumor cells and the tumor microenvironment significantly impact the clinical efficacy of PD-1/PD-L1 immune checkpoint inhibitors [40]. Therefore, a comprehensive understanding of the regulatory mechanisms governing PD-L1 expression across transcriptional, translational, and post-translational levels is crucial. Recent studies have elucidated various mechanisms controlling PD-L1 abundance, such as transcriptional regulation by c-Myc in acute T-lymphocytic leukemia and IRF1 in endometrial cancer [41]. Zhang et al. demonstrated that PD-L1 is a direct proteolytic substrate of SPOP in prostate cancer cells [42]. In our study, we propose a model where SPOP downregulates PD-L1 expression by targeting LMNB2 for ubiquitination and degradation, thereby influencing PD-L1 mRNA levels. This suggests that SPOP may regulate PD-L1 expression through multiple layers, including transcriptional, translational, and post-translational mechanisms, in a context-dependent manner. SPOP governs the stability of numerous substrates that can directly or indirectly affect PD-L1 mRNA expression. Other research has indicated that B-type lamin degradation induces substantial changes in chromatin conformation, particularly near the nuclear periphery [43], implying a complex relationship between LMNB2 and PD-L1. Lamin proteins interact with chromatin and contribute to nuclear organization and structure [34]. Specifically, lamins anchor chromatin to the nuclear lamina, influencing chromatin compaction and accessibility, and thus gene expression. A recent study revealed that LMNB1 promotes gene disinhibition by redistributing peripheral heterochromatin into intranuclear heterochromatic foci [44]. Notably, LMNB2 interacts closely with other lamins, LMNA and LMNB1, to maintain chromosome stability and proper chromatin localization [34]. In addition to directly targeting PD-L1, LMNB2 may also influence PD-L1 expression by regulating chromatin accessibility, a hypothesis that warrants further investigation.

**Fig. 7:**
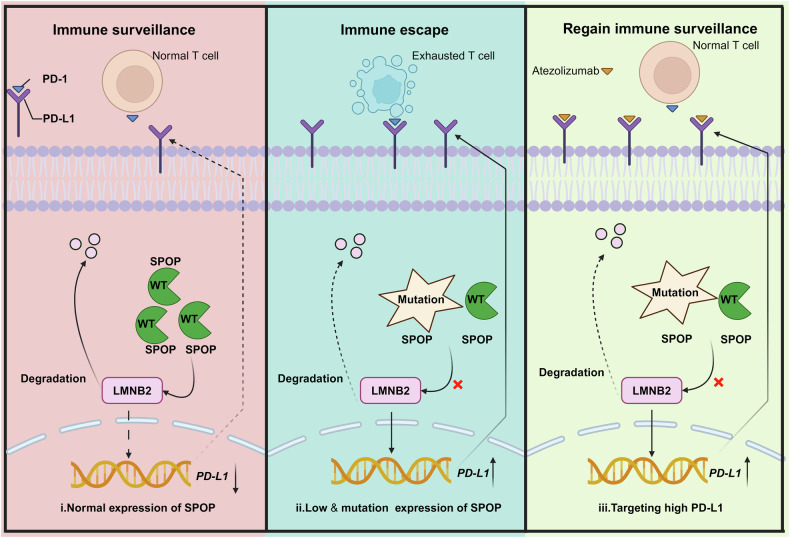
Schematic of the proposed mechanism through which PD-L1 transcription activation induced by SPOP dysfunction induces immune escape of HCC cells.

Previous studies have posited that LMNB2 interacts with tumor immune cells, including B cells, CD8^+^ T cells, CD4^+^ T cells, and macrophages, and may be implicated in HCC progression [45]. In this study, we identified LMNB2 as a novel transcriptional regulator of PD-L1, with a positive relationship with poor prognosis of HCC. This aligns with another study showing consistent expression trends of LMNB2 and PD-L1 in non-small cell lung cancers under various drug treatments [46]. While we have demonstrated that LMNB2 regulates PD-L1 expression by binding to its P6 region (1315–1574) and affecting PD-L1 promoter activity, the specific binding site within LMNB2 remains unclear, necessitating the construction of *LMNB2* mutants to assess their impact on PD-L1 transcriptional activity. Mechanistically, we showed that SPOP downregulates PD-L1 mRNA transcription by targeting LMNB2 for ubiquitination-dependent destruction. However, mutations and pathologically low SPOP expression lead to increased LMNB2 and PD-L1 levels. Notably, while prior studies indicate that SPOP-mediated substrate degradation is proteasome-dependent [23], our findings suggest that SPOP-mediated LMNB2 degradation occurs via the lysosomal pathway, potentially involving autophagy receptors such as p62, NBR1, or OPTN [47]. Furthermore, LMMB2 overexpression enhanced the efficacy of Atezolizumab-based ICB therapy (Fig. 7). Establishing a high-LMNB2-expressing mouse HCC model would strengthen these findings. Additionally, constructing HCC patient-derived organoids could better mimic clinical ICB treatment outcomes. Given LMNB2’s role in HCC immune evasion, developing small-molecule LMNB2 inhibitors via artificial intelligence and high-throughput screening holds significant promise. We also identified an SBC motif in LMNB2; mutations in this motif disrupt the SPOP–LMNB2 interaction. In summary, *SPOP* mutations and low SPOP expression upregulate LMNB2, thereby increasing PD-L1 expression in HCC. Uncovering these genetic mechanisms underlying abnormal PD-L1 expression may guide PD-1/PD-L1 blockade immunotherapy in HCC.

## Material and methods

Antibody, chemicals, primers, and siRNA/sh RNA sequence information used in this study are listed in Tables S1–S3.

### Dual-luciferase reporter assays

Huh7 cells were transiently transfected with Flag-LMNB2 and pCMV-Vector using Lipo6000 Transfection Reagent (Beyotime, Shanghai, China) and a PD-L1 promoter plasmid (full-length and truncated) (MiaoLing, P56701). Forty-eight hours post-transfection, the cells were collected, and measured the activities of both firefly luciferase and renilla luciferase were measured using the dual-luciferase reporter assay system (Vazyme, China, DD1205).

### Plasmid construction and transfections

For overexpression and lentiviral packaging, pCMV-LMNB2-3*Flag, pCMV-Vector, PMD2G, and PSPAX2 were purchased from the MiaoLing plasmid platform. The sources of SPOP-related plasmids have been described previously [48]. LMNB2, SPOP, and PD-L1 promoter plasmid mutants were generated using the KOD-Plus-Mutagenesis kit (TOYOBO) according to the manufacturer’s instructions. All constructs were validated using DNA sequencing.

### Cell culture, transfection, and lentivirus infection

Huh7 (RRID: CVCL_0336) and LO-2 (RRID: CVCL_6926) cells were purchased from Wuhan Pricella Biotechnology Co., Ltd. HEK-293T (RRID: CVCL_0063), HeLa (RRID: CVCL_0030), Jurkat (RRID: CVCL_4530), HepG2 (RRID: CVCL_0027), and Hepa1-6 (RRID: CVCL_0327) cells were purchased from American Type Culture Collection. All cell lines have undergone STR cell line identification and mycoplasma contamination detection as follows: HepG2 (HB-8065), HeLa (CCL-2), HEK-293T (CRL-11268), Jurkat (TIB-152), and Hepa1-6 (CRL-1830) were sourced from the American Type Culture Collection; Huh7 (CL-0120) and LO-2 (CL-0111) were obtained from Wuhan Pricella Biotechnology. HEK-293T, HeLa, Hepa1-6, HepG2, and Huh7 cells were cultured in Dulbecco’s modified Eagle’s medium (DMEM, Meilunbio, China) supplemented with 10% Fetal Bovine Serum (FBS, Standard Quality, OriCell, China). Jurkat cells and LO-2 were cultured in Roswell Park Memorial Institute-1640 (RPMI-1640, Meilunbio, China) with 10% Fetal Bovine Serum (FBS, Standard Quality, OriCell, China). All the cells were grown at 37 °C with 5% CO_2_. The above cell lines were authenticated and checked by short tandem repeat (STR) type. For transfection, cells were transiently transfected with plasmid, siRNA, and shRNA using Lipo6000 Transfection Reagent (Beyotime, Shanghai, China), according to the manufacturer’s protocol. For lentivirus infection, lentivirus plasmids containing pLVX-shNC-Puro negative control, pLVX-shLMNB2-Puro, PLKO.1-shSPOP-Puro, and the corresponding control group lentivirus were purchased from QingKe Biotechnology (Shanghai, China) and were used to infect HepG2 and Huh7 cells according to the manufacturer’s protocol. Infected cells were then subjected to puromycin selection (5 μg/mL) and stable transfection of cells was confirmed by western blotting.

### Western blot

Cells were lysed with RIPA lysis buffer (high) supplemented with protease inhibitors on ice for 30 min. Lysates or immunoprecipitates were subjected to SDS-PAGE and proteins were transferred to nitrocellulose membranes (GE Healthcare, Little Chalfont, UK). Membranes were closed in Tris-buffered saline (TBS, pH 7.4) containing 5% skim milk and 0.1% Tween 20, washed twice in TBS containing 0.1% Tween 20, and incubated overnight at 4 °C with the primary antibody, followed by incubation with the secondary antibody for 1 h at room temperature. The target proteins were visualized using an enhanced chemiluminescence (ECL) system (Santa Cruz Biotechnology). WB was performed 2–3 times from at least two independent experiments, and representative images are shown.

### In vivo ubiquitination assays and co-immunoprecipitation (co-IP)

HEK-293T and Huh7 cells were transfected with HA-ubiquitin and/or the indicated constructs. 36 h after transfection, the cells were treated with MG-132 (20 μM) for 8 h before harvesting, lysed in RIPA lysate (High), and boiled for 10 min. For co-IP, the WCLs were centrifuged at 12,000 rpm for 20 min. The supernatant was removed and incubated with anti-Flag M2 agarose beads (Sigma, USA, RRID: AB_10063035) or recombinant protein G Sepharose beads (Thermo Fisher Scientific, USA) coupled with PD-L1 antibody (RRID: AB_2756526) at 4 °C overnight. The bound beads were then washed three times with BC100 buffer (20 mM Tris–Cl, pH 7.9, 100 mM NaCl, 0.2 mM EDTA, 20% glycerol) containing 0.2% Triton X-100. Proteins were eluted with Flag peptide for 4 h at 4 °C or by boiling in SDS–PAGE solution. The ubiquitinated forms of LMNB2, as well as the immunoprecipitated pull-down proteins, were detected by western blotting using anti-HA antibody, anti-ubiquitin antibody, or anti-LMNB2 antibody coupled with other labeling antibodies.

### Real-time reverse transcription quantitative PCR (RT-qPCR)

Total RNA was isolated from the indicated cells using TRIzol reagent (Tiangen, China), and cDNA was reverse-transcribed using the HiScript® II 1st Strand cDNA Synthesis Kit (Vazyme, China), according to the manufacturer’s instructions. PCR amplification was performed using the SYBR Green PCR Master Mix Kit (Vazyme, China). All quantifications were normalized to the level of the endogenous control GAPDH.

### GST pull-down assay

GST fusion proteins were immobilized on glutathione-Sepharose beads (Amersham Biosciences, USA). The beads were washed using pull-down buffer (20 mM Tris–HCl pH 7.5, 150 mM NaCl, 0.1% NP-40, 1 mM DTT, 10% glycerol, 1 mM EDTA, 2.5 mM MgCl_2_, and 1 μg/mL leupeptin). The beads were incubated with recombinant protein for two hours before being washed five times with binding buffer. Finally, the beads were resuspended in sample buffer, and the bound proteins were subjected to SDS-PAGE and Western blot analysis.

### Cell immunofluorescence

For immunofluorescence (IF), the cells were placed on chamber slides and fixed in 4% paraformaldehyde for 30 min at room temperature. After washing with PBS, cells were permeabilized with 0.1% Triton X-100 in PBS for 15 min. The cells were then washed with PBS, blocked with 0.5% BSA in PBS for 1 h, and incubated with the primary antibody in PBS overnight at 4 °C. The cells were then washed with PBS, blocked with 0.5% BSA in PBS, and incubated with the primary antibody in PBS overnight at 4 °C. After washing with PBS, fluorescently labeled secondary antibodies were applied, and DAPI was re-stained for 1 h at room temperature. Cells were visualized and imaged using a confocal microscope (LEICA TCS SP8).

### Protein half-life assays

For the half-life study, 20 μg/ml of cycloheximide was added to the medium. At the indicated time points, the cells were collected and protein abundance was measured by WB.

### Tissue samples and immunohistochemistry

Clinical data and tissue samples were acquired from the Institutional Review Board of the Ethics of Ningbo University (NBU-2024-313). The informed consent of all the subjects has been obtained. Subject selection criteria: Clinical patients who agreed to participate in the study protocol were regardless of age, sex, and weight. Exclusion criteria: The pathological diagnosis was non-hepatocellular carcinoma. Adverse event: Only liver cancer specimens of patients were taken, and there were basically no adverse events in patients. Patients who underwent radical resection for HCC at Ningbo University’s Affiliated Lihui Li Hospital provided the HCC specimens used in this study. HCC paraffin sections were prepared in ethylenediaminetetraacetic acid (EDTA) antigen repair buffer (pH 9.0) for 8–12 min using a microwave oven. Tissues were treated with a peroxidase-blocking agent (3% hydrogen peroxide) for 30 min. Subsequently, they were rinsed thrice with phosphate-buffered saline with Tween (PBST) solution. Following this, the tissues were blocked using 10% donkey serum and then incubated with the primary antibody at 4 °C overnight. Following three rinses with PBST, the tissue sections were incubated with horseradish peroxidase (HRP)-conjugated secondary antibody for 1 h. Following triple rinsing with PBST, the HCC paraffin sections were stained using a DAB assay kit (#G1212; Solarbio, Beijing, China). Quantitative analysis by Immunohistochemistry (IHC) was conducted using ImageJ (RRID:SCR_003070) and an IHC profiler (a plugin for the quantitative analysis of immunohistochemical samples; https://sourceforge.net/projects/ihcprofiler/). The percentage of positively stained cells was scored as follows: 1. <25%; 2. 25–50%; and 3. >50%. The staining intensity was graded as follows: 0 (no or weak staining = light yellow), 1 (moderate staining = yellowish brown), and 2 (strong staining = brown). The total score for LMNB2 and PD-L1 expression was multiplied by the percentage of cells scored against positive staining and the intensity of the staining score, and a total score ranging from 0 to 6 was assigned.

### Cell proliferation assay

The cell proliferation rate was determined using the Cell Counting Kit-8 (CCK-8) (Dojindo Laboratories, Japan), according to the manufacturer’s protocol. Briefly, cells were inoculated into 96-well plates at a density of 1000 cells per well. During the incubation period of 0–6 days, 10 μl of the CCK-8 solution was added to the cell cultures and incubated for 2 h. The optical density (OD) of each well was measured at 450 nm using a microplate absorbance reader (Bio-Rad, USA). Each assay was performed in triplicate.

### Colony formation assay

HepG2 and Huh7 cells were seeded in triplicate in six-well plates containing 1500 cells per well. After 2 weeks of incubation, the cells were fixed in 100% methanol for 5 min at room temperature and stained with Giemsa dye for 20 min (Solarbio, China).

### Wound-healing assay

HepG2 and Huh7 cells were seeded in six-well plates (Costar, Corning, USA) and cultured to 80% confluence. The monolayers of cells were damaged by removing the culture insert and rinsed with PBS to remove cellular debris. After treatment with Mitomycin C for 1 h (5 μM) (GLPBIO, #GC12353, CA, USA), the medium was replaced with fresh serum-free DMEM. Images were acquired using a fluorescence microscope (Nikon Ds-Ri2, Tokyo, Japan) at 0 and 48 h after migration. The wound-edge healing area was calculated between 0 and 48 h.

### Migration and invasion assay

HepG2 and Huh7 cells were pre-cultured in serum-free medium for 48 h. For the migration assay, 4 × 10^4^ cells were inoculated into the upper side of a modified Boyden chamber (8.0 μm, #3342, Corning, NY, USA), and the lower chamber was filled with medium containing 5% FBS. After 24 h, non-migrating cells were carefully removed from the upper chamber with a cotton swab, stained, and counted in nine different areas below the filter. Matrix gel invasion assays were performed using migration inserts (Costar) coated with matrix gel/fibronectin (BD Biosciences, USA). Images of the stained cells were taken under a microscope (magnification: ×200).

### In vivo xenograft assay

C57BL/6J mice (RRID: IMSR_JAX:000664, female, weighing 15–25 g) were obtained from Beijing Vitonglihua Experimental Animal Technology Co., LTD, for in vivo xenograft experiments. After the constructed stable cell lines were prepared, 50μ PBS and 50μ matrix glue (cat#082704; Mogengel, Xiamen, China) were added for resuspension. The tumor volume was measured every four days and ended at 20 days, starting at 4 days post-transplantation, and calculated using the following formula: tumor volume = (long × wide^2^)×1/2. At the end of the experiment, the tumors were imaged and weighed after the mice were euthanized. Four mice were used in each group and no blinding method was used. All animal procedures were performed according to the protocols approved by the Animal Care Committee of Ningbo University (NBU20240311).

### HCC and Jurkat cells co-culture system

HepG2 and Huh7 cells with stable LMNB2 and/or SPOP knockdown or overexpression were seeded at 1 × 10^5^ cells/well in 12-well plates and transfected with plasmids or siRNAs. After 24 h, the medium was replaced with fresh medium. Jurkat cells were pre-activated for 24 h with 2 μg/mL of soluble human CD3/CD28 T cell activator (Proteintech, #KMS310, Wuhan, China). Jurkat cells were then co-cultured with HepG2 and Huh7 cells, as well as control cells, at a density of 5 × 10^5^. DMSO or Atezolizumab (10 ng/ml) was added to the co-culture. After 24 h, the culture supernatant was collected for ELISA to measure IL-2, IL-4, IL-10, and IFN-γ levels, and Jurkat cells were harvested for flow cytometry.

### Flow cytometry

Cell apoptosis and cell cycle analyses were performed using flow cytometry. The Annexin V-FITC/PI Apoptosis Kit (Multi Sciences, #AP101, Hangzhou, China) was used for apoptosis detection. Cell cycle analysis was performed using a FITC/PI Cell Cycle Staining Kit (Multi Sciences, #CCS012, Hangzhou, China). Both procedures were performed according to the manufacturer’s protocol. The resulting data were subsequently examined using FlowJo software (RRID:SCR_008520).

### PD-L1/PD-1 binding assay

A total of 1 × 10^6^ cells were incubated with 5 μg/ml recombinant human PD-1 FC chimeric protein (#1086-PD-050, R&D Systems, USA) for 30 min at room temperature. Subsequently, the cells were washed with staining buffer and further incubated with an anti-human Alexa Fluor 488 dye-conjugated antibody (Thermo Fisher Scientific, USA) for an additional 30 min at room temperature. After another wash with staining buffer, the cells were subjected to flow cytometry analysis. Flow cytometry data were analyzed using FlowJo software (RRID:SCR_008520), with the relative positive percentage cut-off value established at the median of the maximum signal.

### ELISA cytokine assay

The analysis was conducted using ELISA kits (Multi Sciences, Hangzhou, China) for the following human cytokines: IL-2 (#EK102-48), IL-4 (#EK104/2-48), IL-10 (#EK110/2-48), and IFN-γ (#EK180-48).

### Statistical analysis

Statistical calculations were performed using GraphPad Prism software (v8.0), and images were analyzed and quantified using ImageJ software (RRID:SCR_003070). All data are shown as the mean ± SD of experiments repeated at least three times. The differences between the two groups were analyzed using Student’s *t*-test, and multiple comparisons were performed using two-way analysis of variance (ANOVA). *p < 0.05, **p < 0.01, ***p < 0.001, ****p < 0.0001, no significance (ns) *p* ≥ 0.05.

## Supplementary information

Supplementary Figures and Table

original WB for Figs

original WB for supplement Figs

R script for Figure1D-M

## Data Availability

For The Cancer Genome Atlas (TCGA) database (https://portal.gdc.cancer.gov/), we extracted and downloaded a LIHC dataset using R software (version 4.2.1) and performed differential expression analysis, clinicopathological characterization, prognostic analysis, ROC curve analysis, and correlation analysis. For the GEO database (http://www.ncbi.nlm.nih.gov/geo/), we extracted and downloaded the HCC dataset (GSE76427 and GSE45627) using R software (version 4.2.1) and performed differential expression analysis on the mRNA transcript data of LMNB2 contained in this dataset. RNA-seq data related to drug resistance in liver cancer were obtained from the GEO database (GSE211850, GSE182593, GSE151412, and GSE192771). In addition, single-cell RNA sequencing (sc-RNA seq) data analysis was performed using GEO datasets (GSE238264 and GSE235057).

## Code availability

The analysis of the scRNA-seq dataset GSE238264 (https://www.ncbi.nlm.nih.gov/geo/query/acc.cgi?acc=GSE238264) was conducted using the code provided by Zhang et al. [49], which is accessible at https://github.com/ltkagohara/HCC_cabonivo_visium. For dataset GSE235057 (https://www.ncbi.nlm.nih.gov/geo/query/acc.cgi?acc=GSE235057), the analysis code is detailed in Supplementary File 1.
