# Supplementary material for: LMNB2-mediated high PD-L1 transcription triggers the immune escape of hepatocellular carcinoma
Source: Cell Death Discov. 2025 Jun 7;11:269. doi: 10.1038/s41420-025-02540-7 (PMC12145441; doi:10.1038/s41420-025-02540-7)
Supplement: Supplementary file 1 — Supplementary Figures and Table [file 41420_2025_2540_MOESM1_ESM.pdf]

LMNB2-mediated high PD-L1 transcription triggers the immune escape of hepatocellular carcinoma

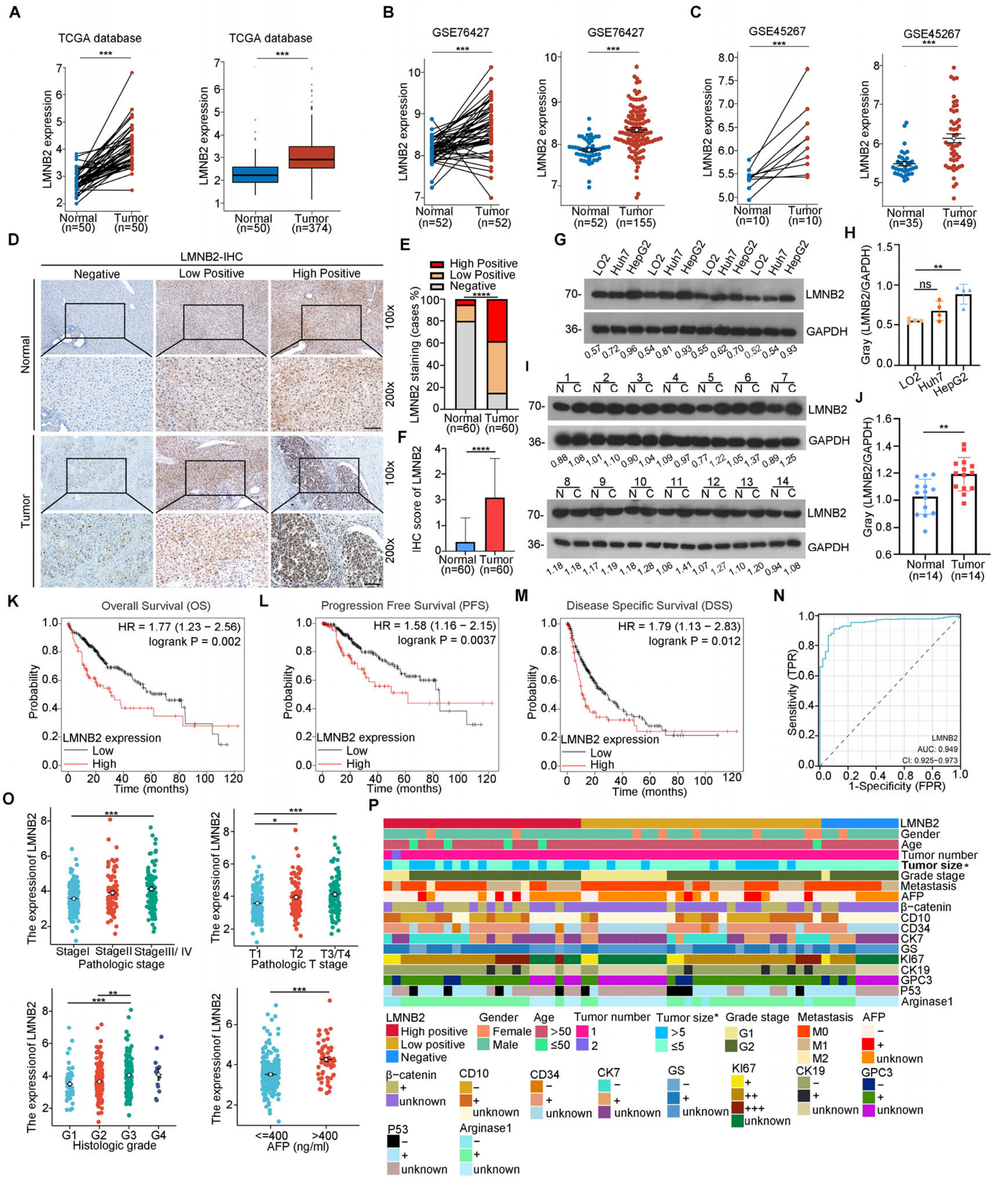

Fig. S1 Elevated LMNB2 expression is associated with poor prognosis in HCC

(A). Analysis of TCGA datasets revealed that LMNB2 mRNA expression was elevated in HCC samples compared to normal liver tissue (normal, n = 50; tumor, n = 374). This trend was also observed when comparing HCC samples with their corresponding paired normal liver tissues

(normal, n = 50; tumor, n = 50). (B). Analysis of the GEO dataset (GSE76427) revealed elevated LMNB2 mRNA expression in HCC samples compared with in that normal liver tissue. This observation was consistent in both unpaired (Normal, n = 52; Tumor, n = 155) and paired (Normal, n = 52; Tumor, n = 52) sample comparisons. (C). Analysis of the GEO dataset (GSE45267) revealed elevated LMNB2 mRNA expression in HCC samples compared with in that normal liver tissue. This observation was consistent in both unpaired (Normal, n = 35; Tumor, n = 49) and paired (Normal, n = 10; Tumor, n = 10) sample comparisons. (D-E). IHC analysis revealed characteristic images showing varied expression of LMNB2 in HCC tissues. The results were categorized into three distinct staining intensities: Negative, Low-positive, and High-positive. Scale bar, 200 μm. Additionally, a statistical graph was presented to illustrate the distribution of these different staining intensities, comparing the normal and tumor tissues. (F). Statistical analysis of the IHC score for LMNB2 in normal and tumor samples. (G-H). Protein expression of LMNB2 in LO2, Huh7, and HepG2 cells. In each group of normal liver cells (LO2), quantification was standardized to GAPDH protein levels. (I-J). WB analysis was performed to examine LMNB2 expression in freshly obtained HCC and normal liver samples. The study included 14 normal (N) and 14 tumor (T) tissue specimens. For each patient, LMNB2 protein levels in the tumor tissues were normalized to GAPDH expression. (K-M). Kaplan-Meier Plotter database analysis revealed the predictive significance of LMNB2 in HCC. (N). The ROC curve shows the predictive value of LMNB2 in identifying HCC tissues based on the TCGA database. (O). Correlation between LMNB2 expression and clinical features of HCC patients in TCGA database. (P). Visualization of clinical features in 60 patients with HCC using a heat map. Data are shown as mean ± SD (n ≥ 3). *$p < 0.05$, **$p < 0.01$, ***$p < 0.001$, ****$p < 0.0001$, ns $p \geq$

0.05.

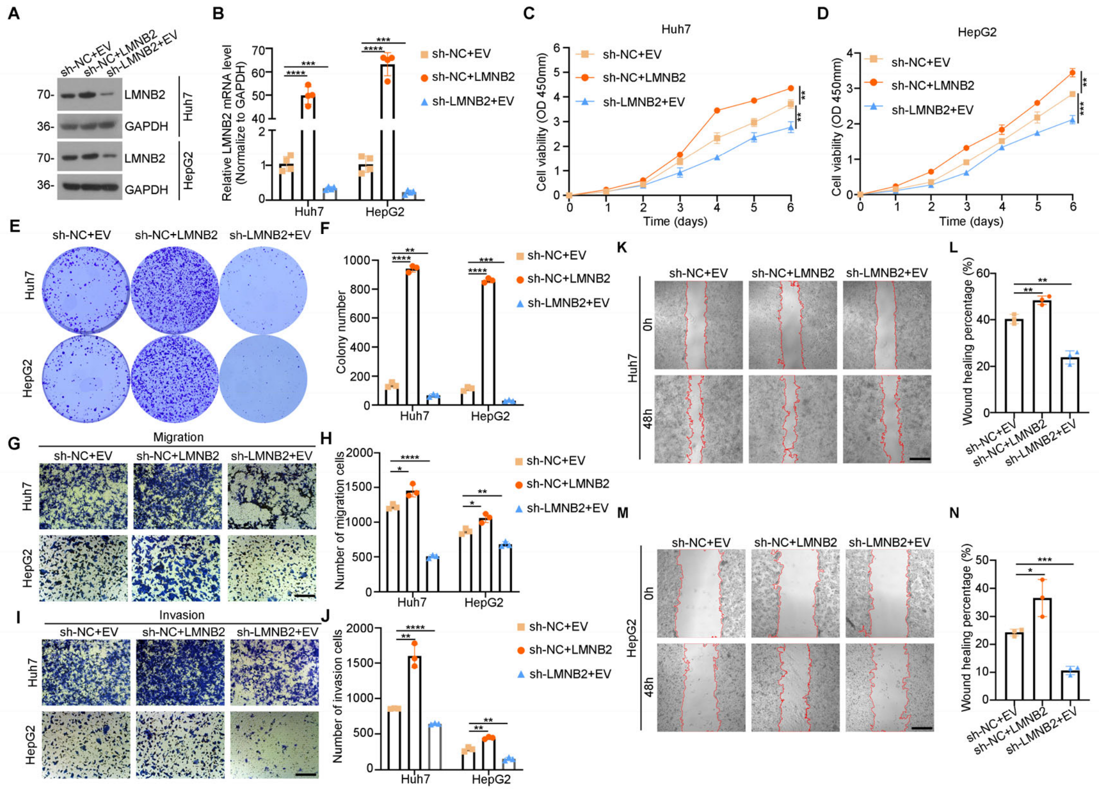

Fig. S2 Elevated LMNB2 promote the malignant phenotypes of HCC cells

(A-B). To assess LMNB2 expression in HCC cell lines Huh7 and HepG2 cells treated with sh-NC+EV, sh-NC+LMNB2, and sh-LMNB2+EV, WB and RT-qPCR were conducted. (C-D). The proliferation of the above treated HCC cells was measured using the CCK-8 assay. (E-F). The proliferation of the above treated HCC cells was measured using colony formation assays. (G-H). The migration of above treated HCC cells was assessed using a transwell assay. Scale bar, 500 μm. (I-J). Invasion of the above treated HCC cells was assessed using a transwell assay. Scale bar, 500 μm. (K-L). Wound healing assay of above treated Huh7. Scale bar, 500 μm. (M-N). Wound-healing assay of above treated HepG2 cells. Scale bar, 500 μm. Data are shown as mean ± SD (n ≥ 3). *$p < 0.05$, **$p < 0.01$, ***$p < 0.001$, ****$p < 0.0001$.

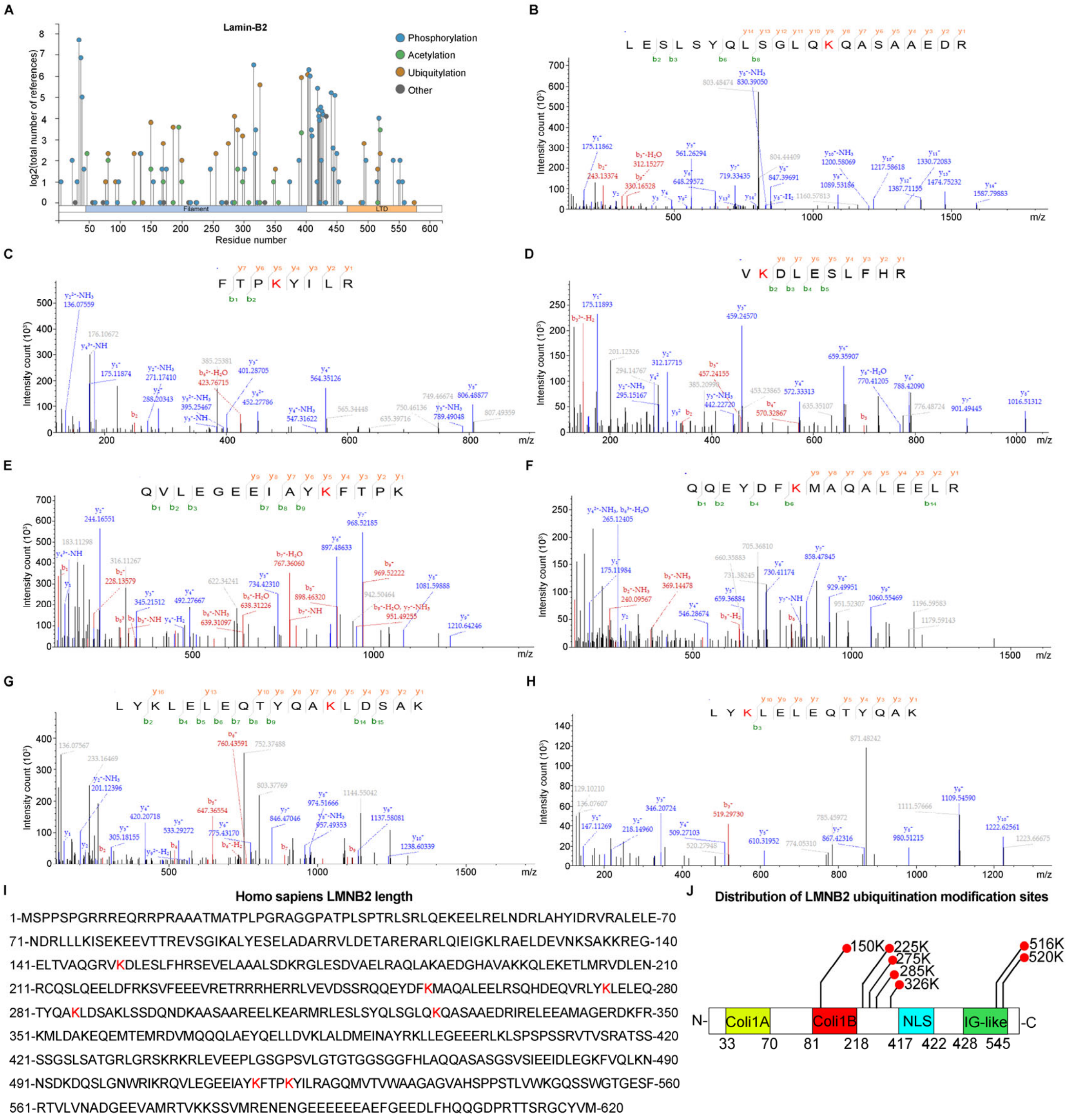

Fig. S3 Identification of LMNB2 ubiquitination modification sites mediated by SPOP

(A). The overview of post-translational modification sites of LMNB2 in the PhosphoSitePlus database. (B-H). Identification of ubiquitination modification sites in LMNB2 mediated by SPOP. (I-J). The distribution of ubiquitination modification sites of LMNB2 mediated by SPOP.

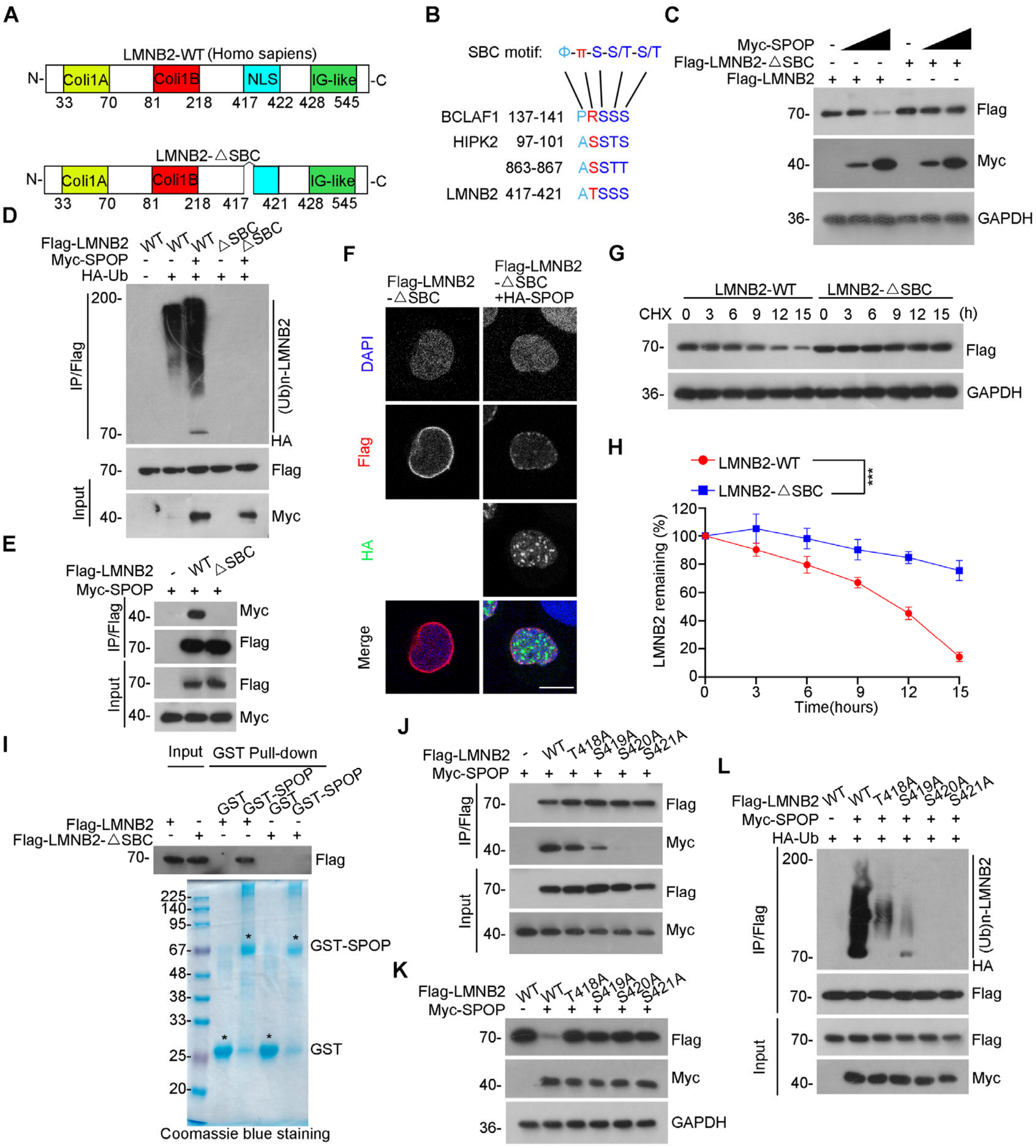

Fig. S4 Identification of the SBC motif in LMNB2 function as a degron recognized by SPOP (A-B). Founding a potential SBC and deletion mutants of LMNB2: SBC (417-ATSSS-421). (C). Effect of SPOP on LMNB2-WT and LMNB2-ΔSBC degradation (D). Effects of SPOP on the ubiquitination of LMNB2-WT and LMNB2-ΔSBC. (E). Interaction between SPOP and LMNB2-WT or LMNB2-ΔSBC. (F). Co-localization between SPOP and LMNB2-WT or LMNB2-ΔSBC. Scale bar, 50 μm. (G-H). Effect of SPOP on LMNB2-ΔSBC half-life (I). *In vitro* interactions between SPOP and LMNB2-WT or LMNB2-ΔSBC (J). Interaction between SPOP and LMNB2-

WT, LMNB2-T418A, LMNB2-S419A, LMNB2-S420A or LMNB2-S421A. (K). Effects of SPOP on degradation of LMNB2-WT, LMNB2-T418A, LMNB2-S419A, LMNB2-S420A, and LMNB2-S421A. (L). Effects of SPOP on ubiquitination of LMNB2-WT, LMNB2-T418A, LMNB2-S419A, LMNB2-S420A, and LMNB2-S421A. Data are shown as mean ± SD (n ≥ 3). ***$p < 0.001$.

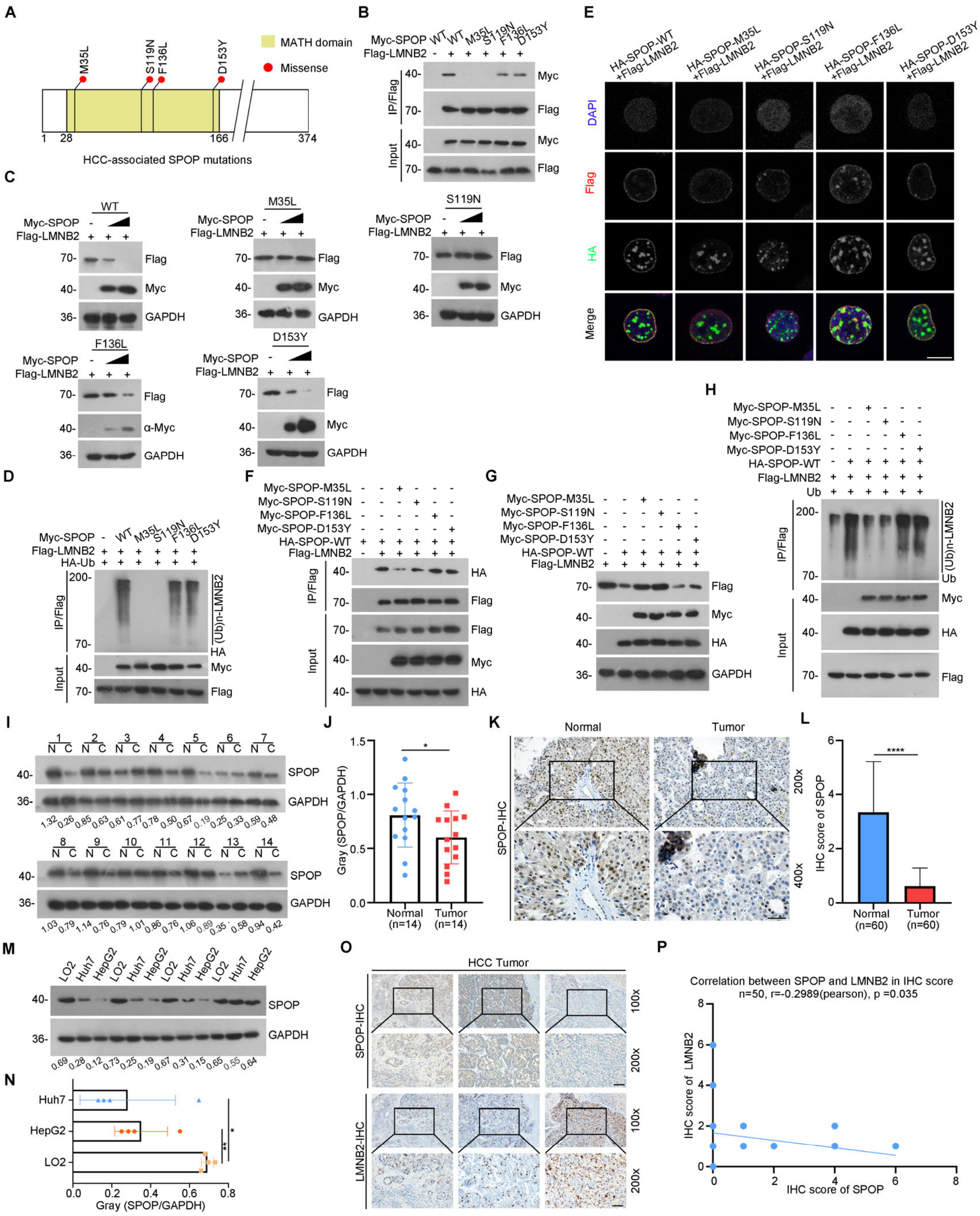

Fig. S5 HCC-associated mutations or low expression of SPOP failing to mediate the

ubiquitination and degradation of LMNB2

(A). Distribution of point mutations in the SPOP gene in HCC specimens. (B). The interaction between SPOP-WT, SPOP-M35L, SPOP-S119N, SPOP-F136L or SPOP-D153Y and LMNB2-WT. (C). The effects of SPOP-WT, SPOP-M35L, SPOP-S119N, SPOP-F136L and SPOP-D153Y on degradation of LMNB2-WT. (D). The effects of SPOP-WT, SPOP-M35L, SPOP-S119N, SPOP-F136L or SPOP-D153Y on ubiquitination of LMNB2-WT. (E). The co-localization between SPOP-WT, SPOP-M35L, SPOP-S119N, SPOP-F136L or SPOP-D153Y and LMNB2-WT. Scale bar, 50 μm. (F). Effect of SPO-M35L, SPO-S119N, SPO-F136L or SPO-D153Y on the interaction between SPOP-WT and LMNB2-WT. (G). Effect of SPO-M35L, SPO-S119N, SPO-F136L or SPO-D153Y on the degradation of LMNB2-WT by SPOP-WT. (H). Effect of SPO-M35L, SPO-S119N, SPO-F136L, or SPO-D153Y on ubiquitination of LMNB2-WT by SPOP-WT. (I-J). WB analysis was performed to examine SPOP expression in freshly obtained HCC and normal liver samples. The study included 14 normal (N) and 14 tumor (T) tissue specimens. For each patient, LMNB2 protein levels in the tumor tissues were normalized to GAPDH expression. (K). Representative images of SPOP staining in normal liver and liver cancer tissues. Scale bar, 200μm. (L). Statistical analysis of the IHC score of SPOP between normal and tumor samples. (M-N). SPOP protein expression in LO2, Huh7, and HepG2 cells. In each group of normal liver cells (LO2), quantification was standardized to GAPDH protein levels. (O). IHC staining of SPOP and LMNB2 in HCC samples. Scale bar, 200 μm. (P). Correlation analysis between SPOP and LMNB2 IHC scores. Data are shown as mean ± SD (n ≥ 3). *$p < 0.05$, **$p < 0.01$, ****$p < 0.0001$.

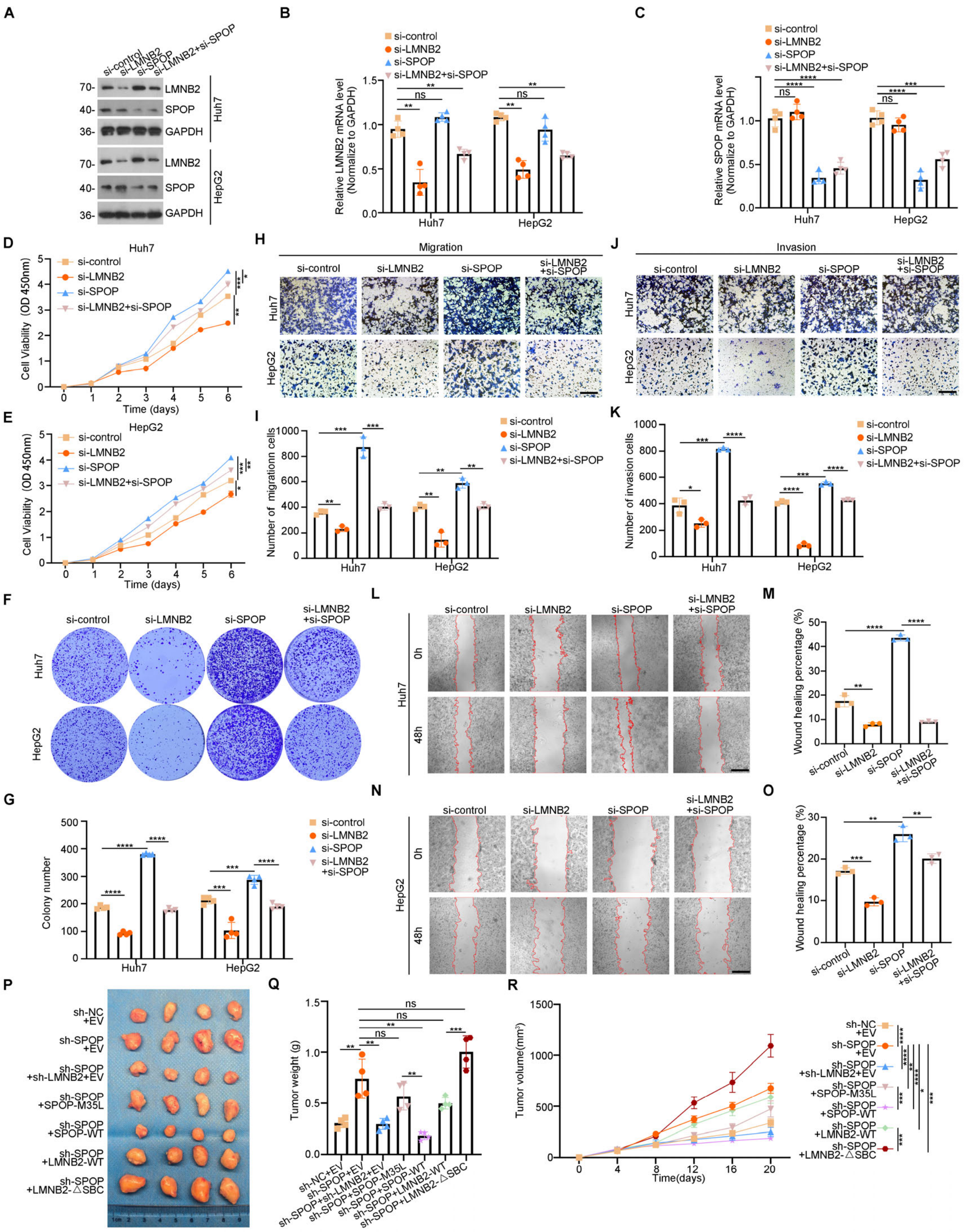

Fig. S6 SPOP can partly reverse the effect of LMNB2 on HCC cells of malignant phenotypes (A-C). To assess SPOP and LMNB2 expression in HCC cell lines Huh7 and HepG2 cells treated with si-control, si-LMNB2, si-SPOP, and si-LMNB2+si-SPOP, WB and RT-qPCR were conducted. (D-E). The proliferation of the treated HCC cells was measured using the CCK-8 assay. (F-G). The proliferation of the treated HCC cells was measured using colony formation

assays. (H-I). The migration of above treated HCC cells was assessed using a transwell assay. Scale bar, 500 μm. (J-K). Invasion of above treated HCC cells was assessed using a transwell assay. Scale bar, 500 μm. (L-M). Wound healing assay of above treated Huh7. Scale bar, 500 μm. (N-O). Wound-healing assay of above treated HepG2 cells. Scale bar, 500 μm. (P). Schematic representation of xenograft tumors of sh-NC+EV, sh-SPOP+EV, sh-SPOP+sh-LMNB2+EV, sh-SPOP+SPOP-M35L, sh-SPOP+SPOP-WT, sh-SPOP+LMNB2-WT, and sh-SPOP+LMNB2-△SBC. (Q). Weight of xenograft tumors in the above groups. (R). Volume of xenograft tumor in the above group. Data are shown as mean ± SD ($n \geq 3$). *$p < 0.05$, **$p < 0.01$, ***$p < 0.001$, ****$p < 0.0001$, ns $p \geq 0.05$.

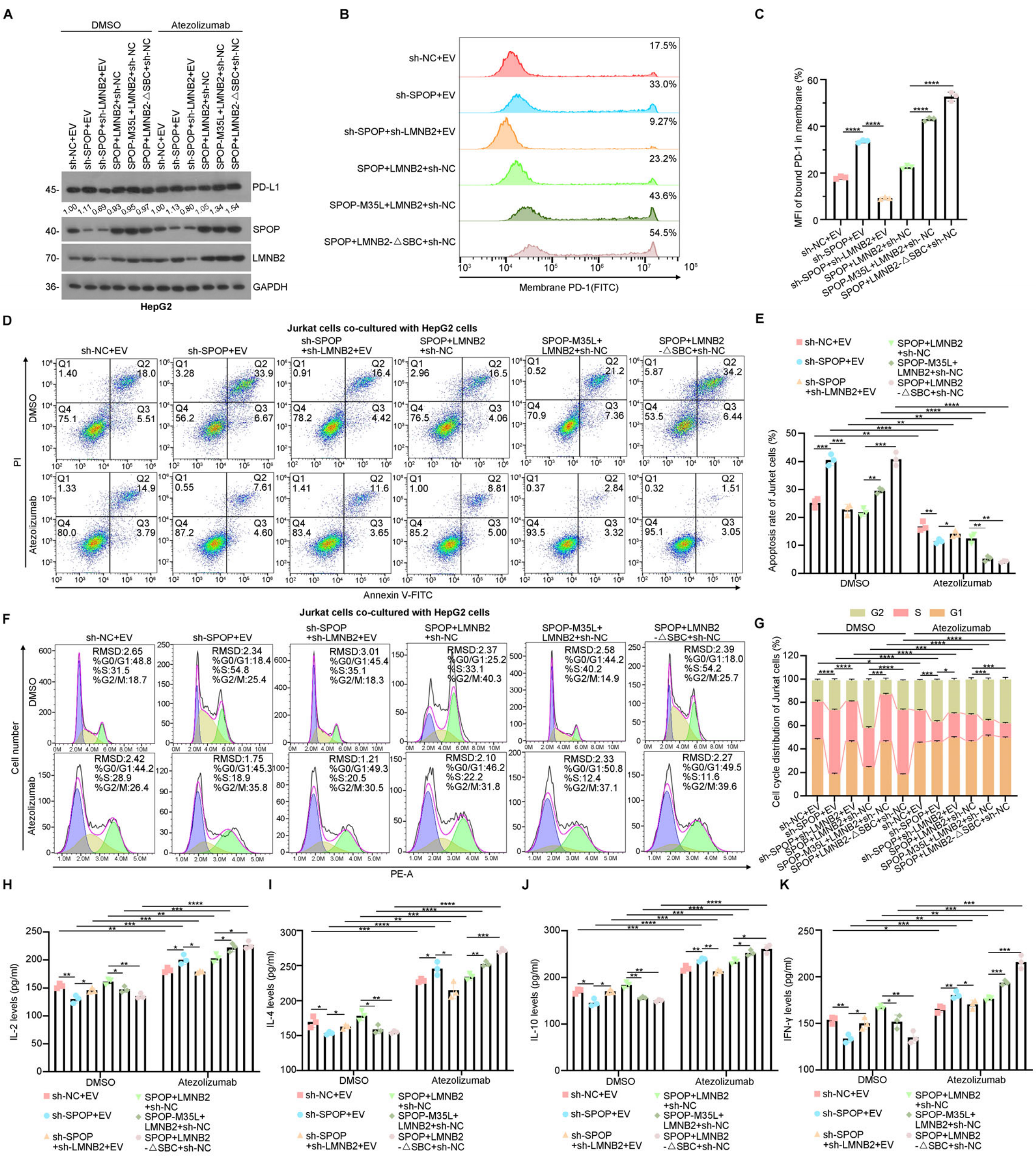

Fig. S7 SPOP-induced immune surveillance depends on LMNB2-PD-L1 axis in HepG2 cell (A). HepG2 cells achieving sh-NC+EV, sh-SPOP+EV, sh-SPOP+sh-LMNB2+EV, SPOP+LMNB2+sh-NC, SPOP+LMNB2-△SBC+sh-NC and SPOP-M35L+LMNB2+sh-NC were co-cultured with Jurkat cells for 24 h after treatment with DMSO or Atezolizumab (10 ng/mL). WB of HepG2 cells in the HCC cell-Jurkat cell co-culture system for detection of PD-L1 expression levels. All quantitation was normalized to the protein level of GAPDH in the sh-NC+EV group. (B). HepG2 cells achieving above treated were co-cultured with Jurkat cells for

24 h. Flow cytometry analysis of PD-1 binding on the HepG2 cell surface. (C). Statistics of the mean fluorescence intensity (MFI) for PD-1 in (B). (D). HepG2 cells achieving above treated were co-cultured with Jurkat cells for 24 h after treatment with DMSO or Atezolizumab (10 ng/ml). Apoptosis levels in Jurkat cells were detected by flow cytometry. (E). Statistical analysis of apoptotic levels in Jurkat cells in (D). (F). HepG2 cells achieving above treated were co-cultured with Jurkat cells for 24 h after treatment with DMSO or Atezolizumab (10 ng/ml). The cell cycles of Jurkat cells were detected by flow cytometry. (G). Cell cycle statistics of Jurkat cells in (F). (H-K). The levels of IL-2, IL-4, IL-10, and IFN-γ produced by Jurkat cells were detected using ELISA. Data are shown as mean ± SD (n ≥ 3). *$p < 0.05$, **$p < 0.01$, ***$p < 0.001$, ****$p < 0.0001$.

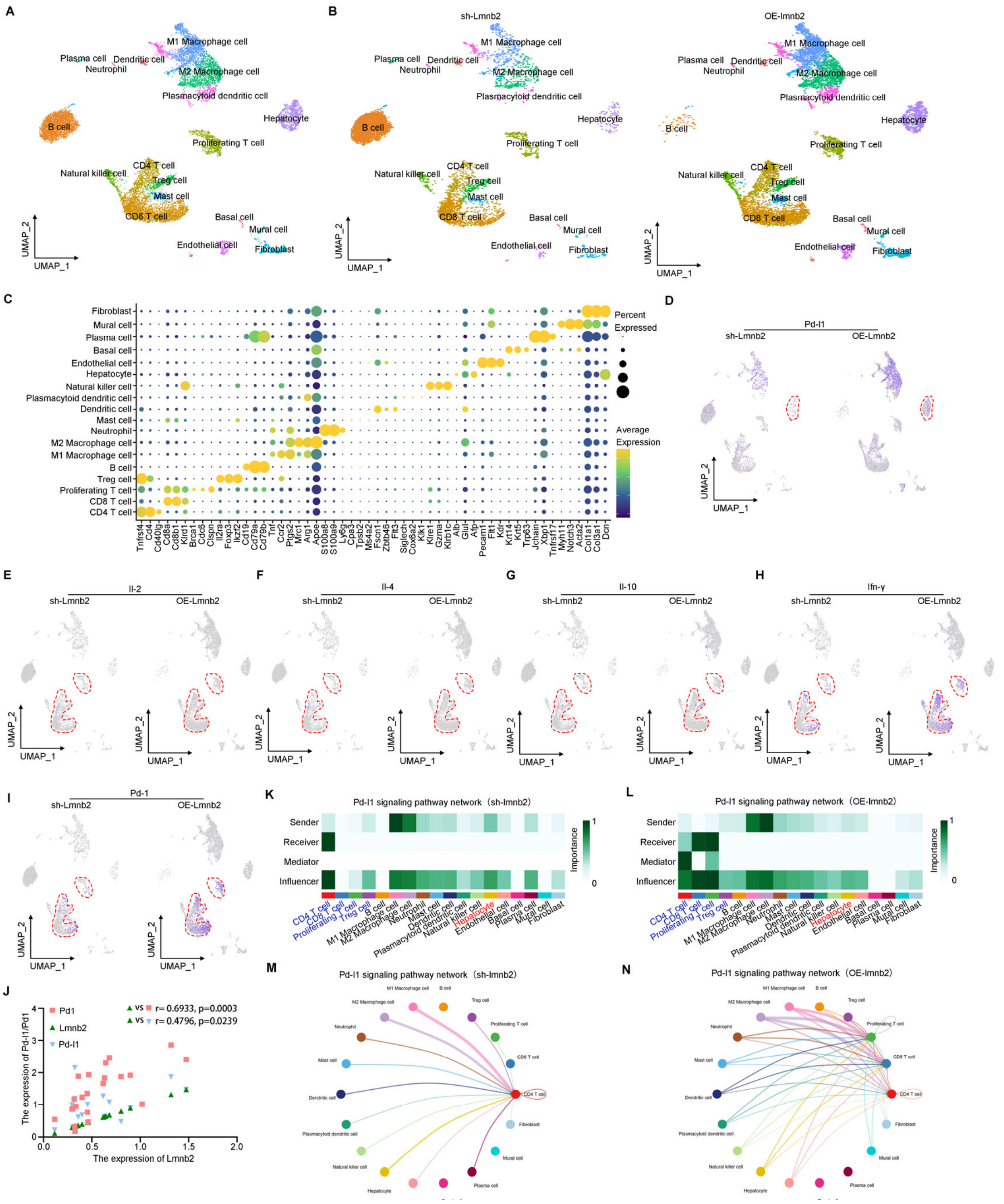

Fig. S8 HCC cells with high Lmnb2 expression can promote communication with T cells partly by Pd-l1 signaling

(A-C). Debatching effect, clustering, and annotation for sc-RNA seq of xenograft tumors grouped sh-Lmnb2 and OE-Lmnb2. (D). The expression of Pd-l1 on HCC cells in sh- Lmnb2 group and OE-Lmnb2 group. (E). The expression of Il-2 on T cells in sh- Lmnb2 group and OE-Lmnb2 group. (F). The expression of Il-4 on T cells in sh- Lmnb2 group and OE-Lmnb2 group.

(G). The expression of Il-10 on T cells in sh- Lmnb2 group and OE-Lmnb2 group. (H). The expression of Ifn-γ on T cells in sh- Lmnb2 group and OE-Lmnb2 group. (I). The expression of Pd-1 on T cells in sh- Lmnb2 group and OE-Lmnb2 group. (J). The correlation analysis of the expression of Lmnb2 and Pd-l1/Pd1 total groups in sc-RNA seq. (K-L). Social network analysis identifies the role of each type of cell in the Pd-1 signaling pathway of sh- Lmnb2 group and OE-Lmnb2 group. (M-N). Visualization of cell interactions (circle plot) in the Pd-1 signaling pathway of sh- Lmnb2 group and OE-Lmnb2 group.

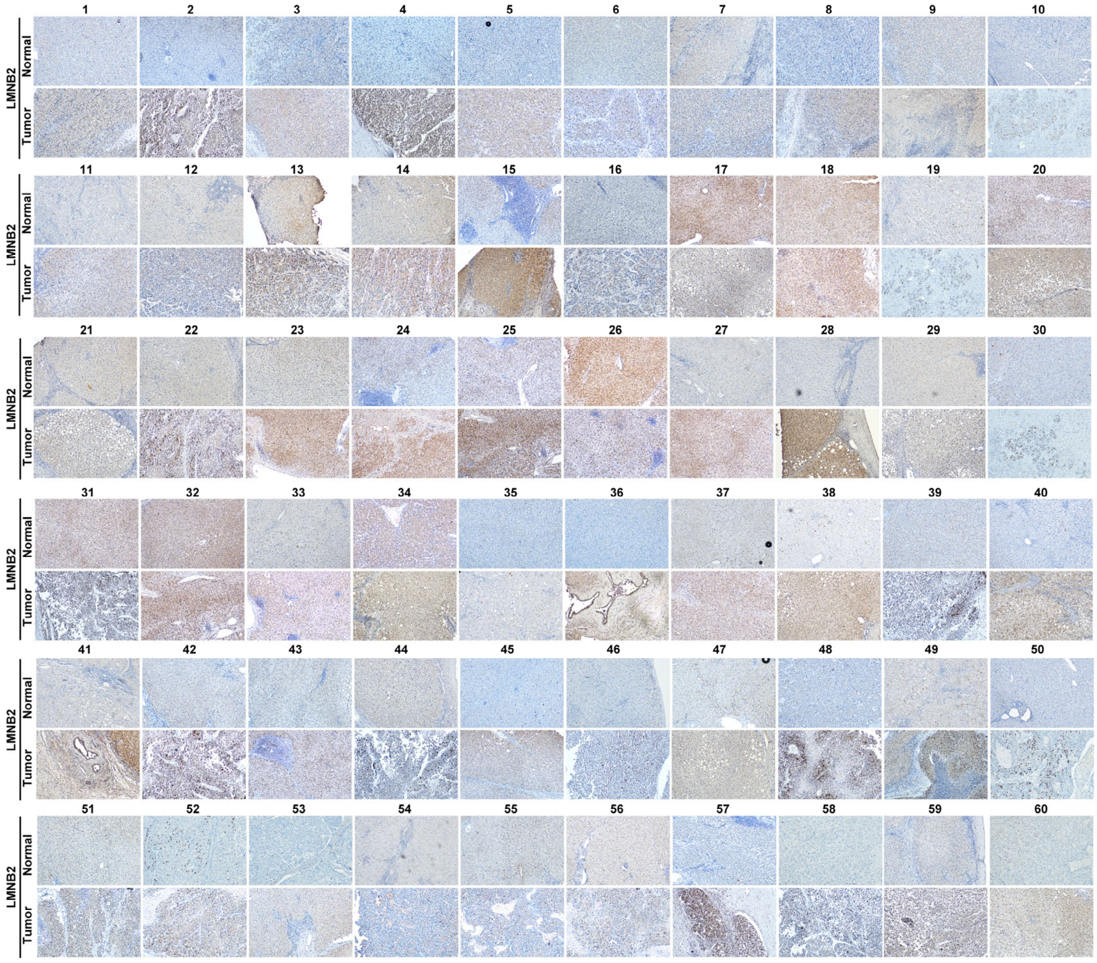

Fig. S9 IHC of LMNB2 in HCC paired samples

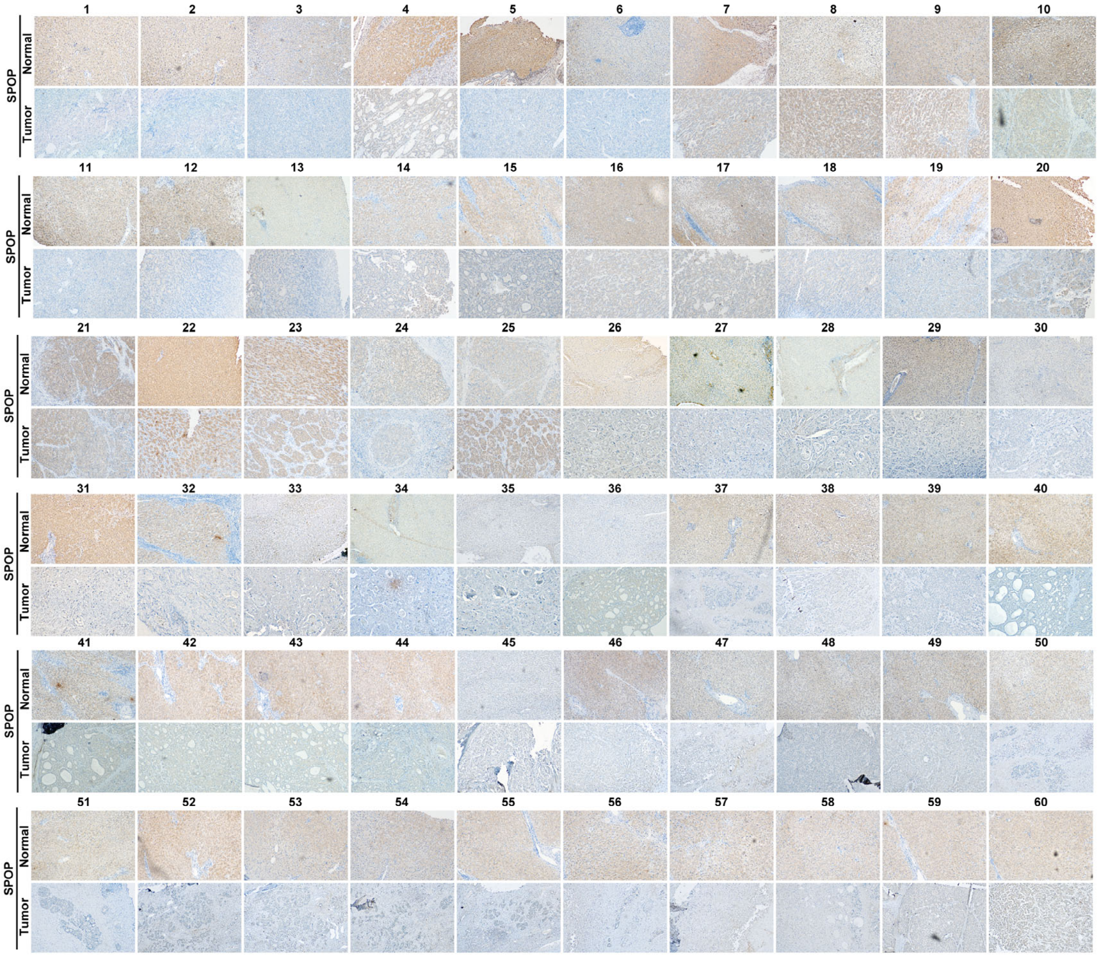

Fig. S10 IHC of SPOP in HCC paired samples

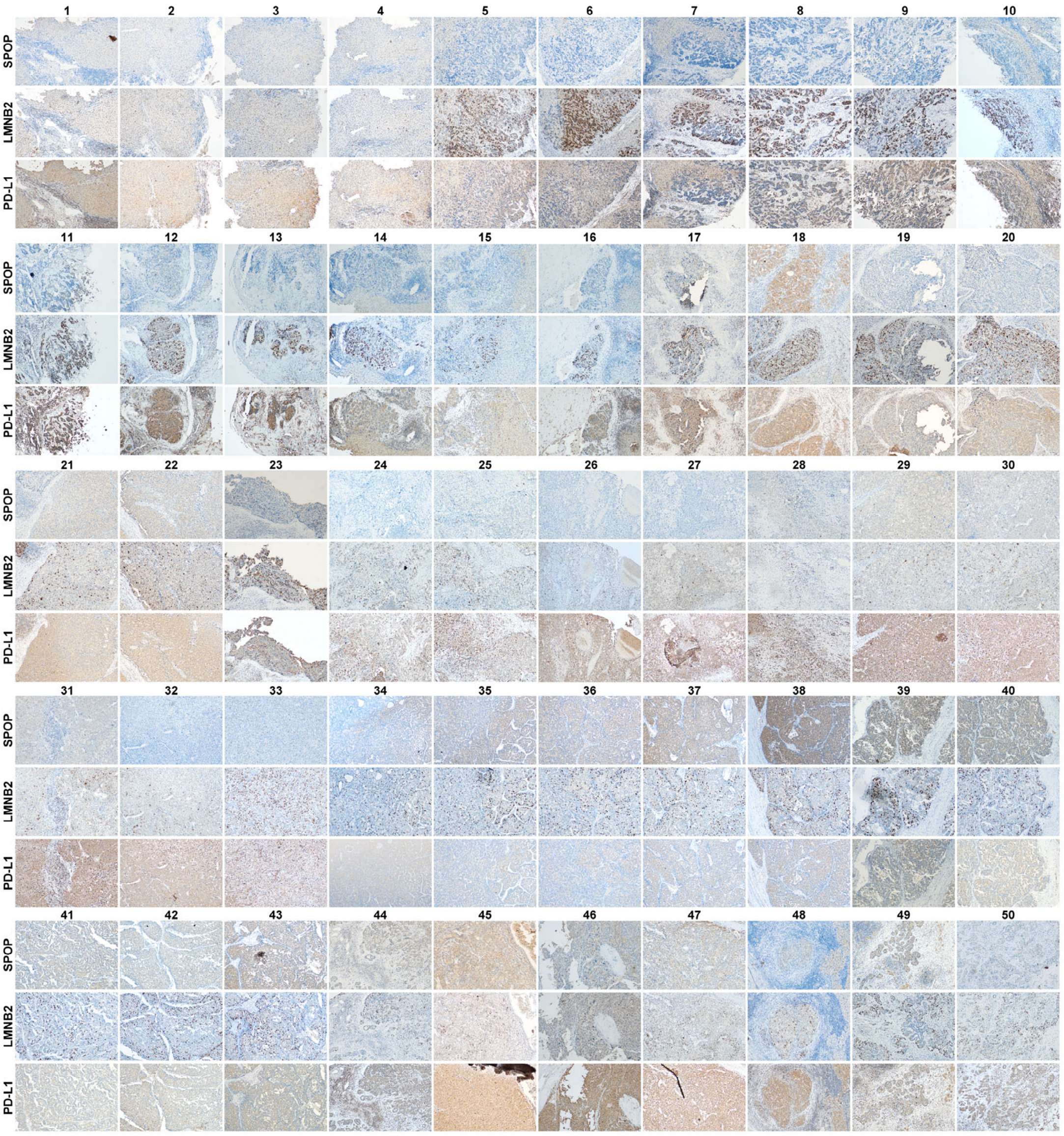

Fig. S11 IHC of SPOP-LMNB2-PD-L1 in HCC paired samples

Table S1. Primer for RT-qPCR and constructions, siRNA and shRNA oligonucleotide sequences

**SYBR ®Green RT-qPCR primers**	
LMNB2	RTF: 5'-TGGAGATCAACGCCTACCG-3'
	RTR: 5'-AGCCGCTTCCGCTTACTG-3'
PD-L1	RTF: 5'-CCTACTGGCATTTGCTGAACGCAT-3'
	RTR: 5'-TTTGCTGAACGCCCCATACA-3'
SPOP	RTF: 5'-CCCGTAGCTGAGAGTTGGTG-3'

	RTR: 5'-CCAGGGCATACTTGTCAGCA-3'
GAPDH	RTF: 5'-GGTGGTCTCCTCTGACTTCAACA-3'<br>RTR: 5'-GTTGCTGTAGCCAAATTCGTTGT-3'
**siRNA and shRNA oligonucleotide sequences**	
si-LMNB2	5'-GCGAGGUGAGUGGCAUCAATT-3'
si-SPOP#1	5'-GGAUGAUGUAAAUGAGCAA-3'
si-SPOP#2	5'-GGGCUUCUCCCUGA UGACAAGCUUA-3'
si-Control	5'-UUCUCCGAACGUGUCACGUTT-3'
sh-LMNB2	5'-GCGAGGUUGGCAUCAATT-3'
sh-SPOP	5'-<br>GGTTAGATGAAGAAAGCAAAGTTCAAGAGACTTTGCTTTC<br>TTCATCTAACC-3'
sh-Control	5'-TTCTCCGAACGTGTCACGT-3'
**construction primers for KOD-Plus Mutagenesis Kit**	
SPOP-ΔMATH-F	5'-TCTGTCAACATTTCTGGCCAGAATA-3'
SPOP-ΔMATH-R	5'-GATCTGTGTGTAGCACCAACTCTCA-3'
SPOP-ΔBTB-F	5'-TCCGTGGAGAACGCTGCAGAAATTC-3'
SPOP-ΔBTB-R	5'-CTCAGGAACCTTTACCATGTTCATG-3'
SPOP-ΔNLS-F	5'-CTCGAGTACCCATACGACGTACCTG-3'
SPOP-ΔNLS-R	5'-AGGGCACTGTGCTGAAGCCAGAGAG-3'
SPOP-M35L-F	5'- CTGTGGACCATCAATAACTTTAGCT-3'
SPOP-M35L-R	5'- GTAGGAGAATTTCACTACCTTGATC-3'
SPOP-S119N-F	5'-AATCAACGGGCATATAGGTTTGTGC-3'
SPOP-S119N-R	5'-CTCCATAGCTTTGGTTTCTTCTCCC-3'
SPOP-F136L-F	5'-TTAATCCGTAGAGATTTTCTTTTGG-3'
SPOP-F136L-R	5'-TTTCTTGAATCCCCAGTCTTTGCCT-3'
SPOP-D153Y-F	5'- TACAAGCTTACCCTCTTCTGCGAGG -3'
SPOP-D153Y-R	5'- ATCAGGGAGAAGCCCGTTGGCCTCA-3'
LMNB2-ΔSBC-F	5'-AGCGGCAGCTTGTCCGCCACCGGGC-3'
LMNB2-ΔSBC-R	5'-TCGTGAGACGGTGACGCGCGAGGAT-3'

LMNB2-T418A-F	5'-GCCGCCTCGAGCAGCAGCGGCAGCTT-3'
LMNB2-T418A-R	5'-TCGTGAGACGGTGACGCGCGAGGAT-3'
LMNB2-S419A-F	5'-GCCACCGCGAGCAGCAGCGGCAGCTT-3'
LMNB2-S419A-R	5'-TCGTGAGACGGTGACGCGCGAGGAT-3'
LMNB2-S420A-F	5'-GCCACCTCGGCCAGCAGCGGCAGCTTGTCCGC-3'
LMNB2-S420A-R	5'-TCGTGAGACGGTGACGCGCGAGGAT-3'
LMNB2-S421A-F	5'-GCCACCTCGAGCGCCAGCGGCAGCTTGTCCGC-3'
LMNB2-S421A-R	5'-TCGTGAGACGGTGACGCGCGAGGAT-3'
PD-L1-promotor-△1-1050-F	5'-AGATCTCGAGCTCAAGCTTCGAATTCAGAAAAAGAGAAAAAAAAGAAAAG-3'
PD-L1-promotor-△1-1050-R	5'-TTTTTGGCATCTTCCATGTGGGATCCGAGATACTGGGCCGTGGGCA-3'
PD-L1-promotor-△1051-2100-F	5'-TCGAGCTCAAGCTTCGAATTCTTTCTCTTTTTCTAAACACAGCCTGT-3'
PD-L1-promotor-△1051-2100-R	5'-GCATCTTCCATGGTGGGATCCAAATTGAGAAATTGGACTCTTCGTT-3'
PD-L1-promotor-△1575-2100-F	5'-GGCATTGCAGATAGTAGATCTAAGT-3'
PD-L1-promotor-△1575-2100-R	5'-GAATTCGAAGCTTGAGCTCGAGATC-3'
PD-L1-promotor-△1051-1574-F	5'-AGAAAAAGAGAAAAAAAAGAAAAGGGAGCAC-3'
PD-L1-promotor-△1051-1574-R	5'-CTCTGATATTTTCATTTAATGTTTTATTTTTCTAAAAATGG-3'
PD-L1-promotor-△1051-1314-F	5'-AGAAAAAGAGAAAAAAAAGAAAAGGGAGCAC-3'
PD-L1-promotor-△1051-1314-R	5'-CAGCTCAGATGTTCCTTCTTTCAAA-3'
PD-L1-promotor-△1315-1574-F	5'-GGCCCAAACCCTATTGCAATTTTAT-3'

PD-L1-promotor-△1315-1574-R	5’-CTCTGATATTTTCATTTAATGTTTTATTTTTCTAAAAATGG-3’

Table S2. Antibody information

Antibody	Cytokine Species	Cat. No	Source	Application/ Dilutions
Anti-PD-L1	Mouse	66248-1-Ig	Proteintech	IB:1:1000<br>IHC:1:250
Anti-LMNB2	Rabbit	10895-1-AP	Proteintech	IB:1:2000<br>IHC:1:250
Anti-SPOP	Rabbit	16750-1AP	Proteintech	IB:1:5000<br>IHC:1:250
Anti-CD3	Rabbit	17617-1-AP	Proteintech	IHC:1:1000
Anti-CD8	Mouse	66868-1-Ig	Proteintech	IHC:1:10000
Anti-PD-1	Rabbit	18106-1-AP	Proteintech	FC
Anti-Ub	Rabbit	10201-2-AP	Proteintech	IB:1:1000
Anti-Flag	Mouse	M185-7	MBL	IB: 1:4000
Anti-Myc	Mouse	M192-7	MBL	IB: 1:4000

Anti-HA	Mouse	M180-7	MBL	IB: 1:4000
Anti-Flag	Mouse	66008-4-Ig	Proteintech	IF: 1:500
Anti-HA	Rabbit	81290-1-RR	Proteintech	IF: 1:500
Anti-GAPDH	Rabbit	AC001	Abclonal	IB: 1:8000
Anti-Mouse	Donkey	AS033	Abclonal	IB:1:8000<br>IHC:1:200
Anti-Rabbit	Donkey	AS038	Abclonal	IB:1:8000<br>IHC:1:200
Anti-Rabbit-488	Donkey	AS035	Abclonal	IF: 1:200
Anti-Mouse-Cy3	Goat	AS008	Abclonal	IF: 1:250
Anti-Flag beads	Mouse	M2	Sigma	IP
Anti-Alexa Fluor 488 dye	Goat	A-11013	Thermo Fisher Scientific	FC

Table S3. Chemicals

Name	Cat. No	Source
Atezolizumab	HY-P9904	MedChemExpress
MG-132	S2619	Selleckchem
CHX	S7418	Selleckchem
Puromycin	S7417	Selleckchem

Mitomycin C	GC12353	GLPBIO
CD3/CD28 T-cell activator	KMS310	Proteintech
