# Supplementary figures and images for: LMNB2-mediated high PD-L1 transcription triggers the immune escape of hepatocellular carcinoma
Source: Cell Death Discov. 2025 Jun 7;11:269. doi: 10.1038/s41420-025-02540-7 (PMC12145441; doi:10.1038/s41420-025-02540-7)

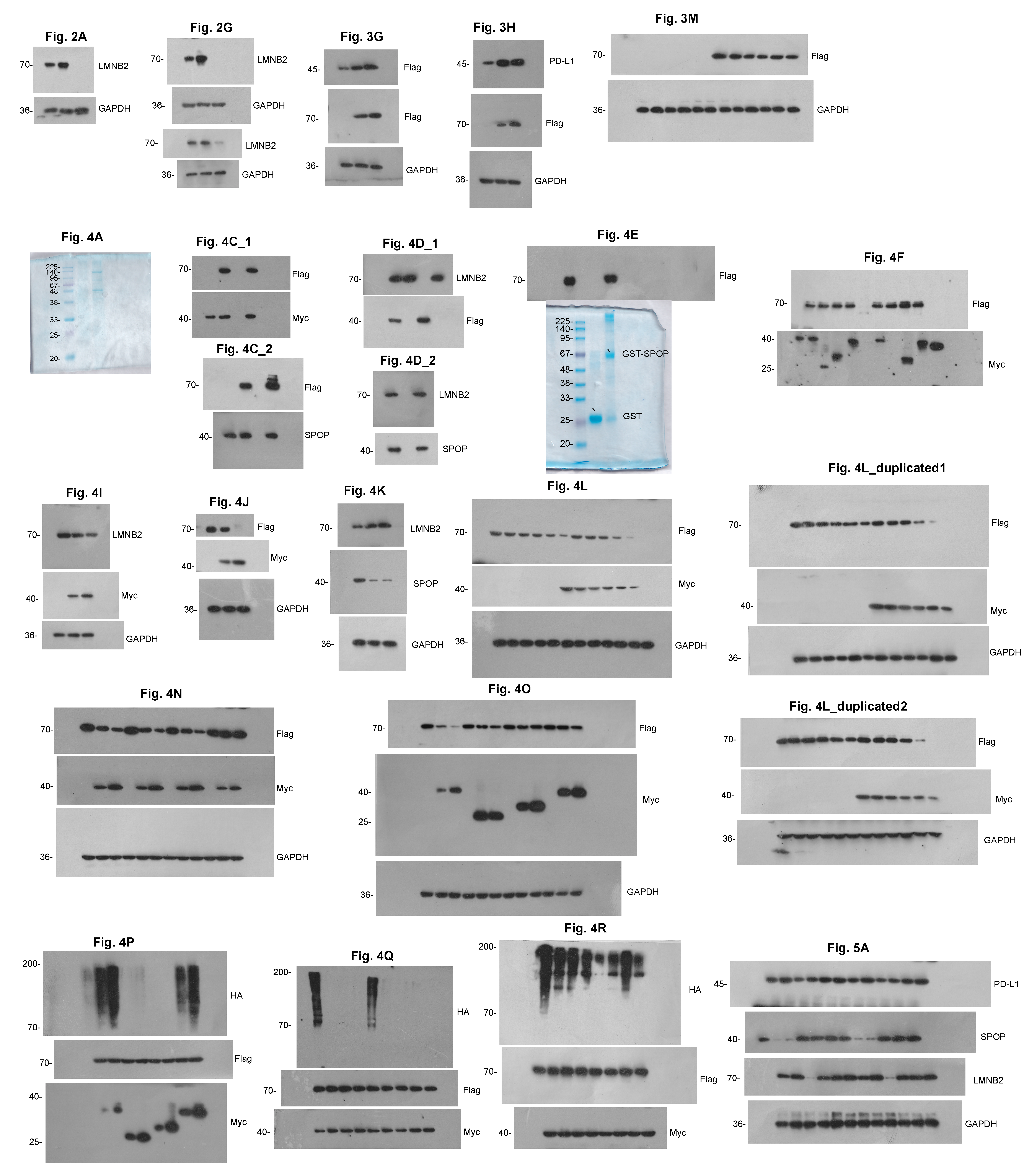

Supplement: Supplementary file 2 — original WB for Figs [file 41420_2025_2540_MOESM2_ESM.png]

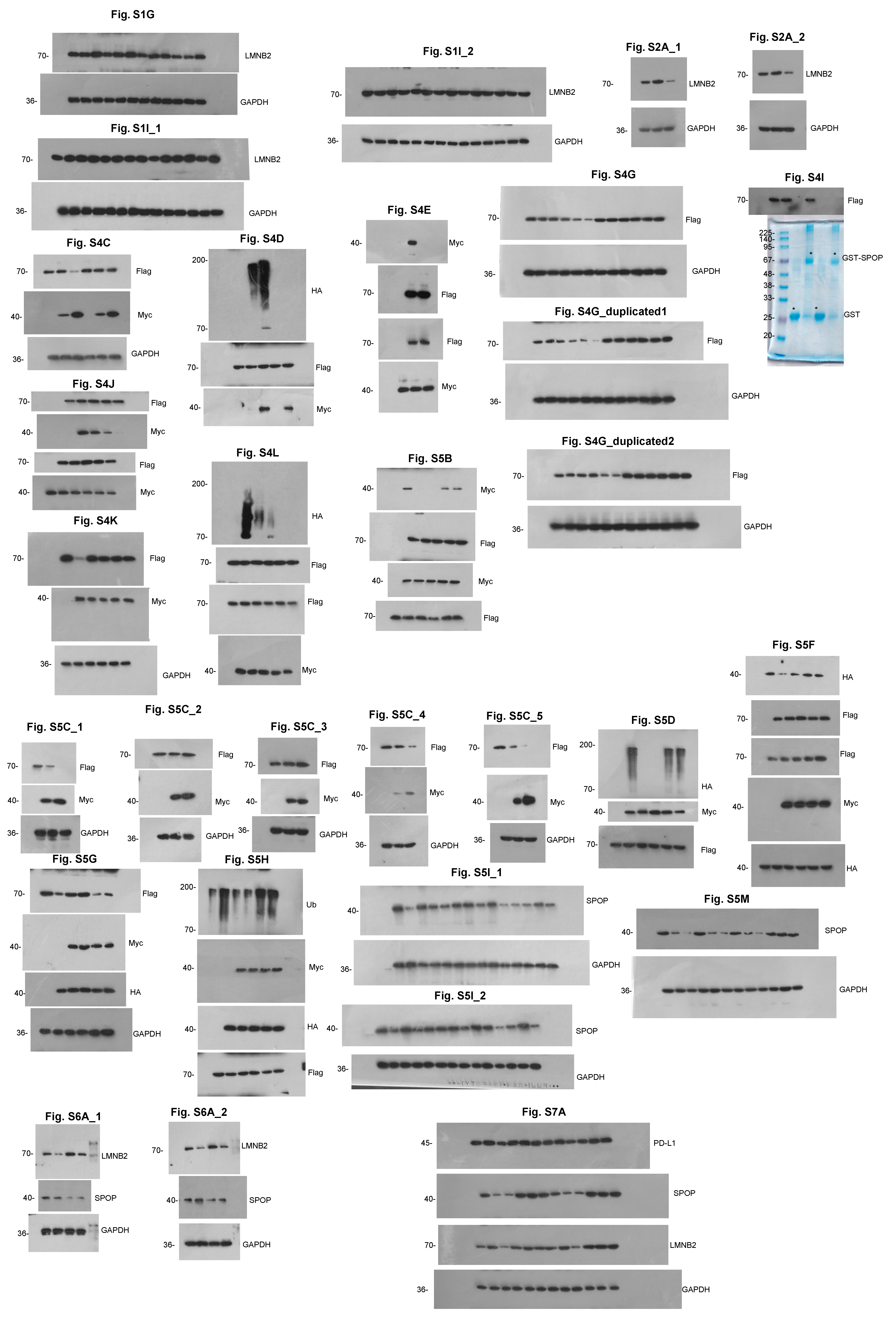

Supplement: Supplementary file 3 — original WB for supplement Figs [file 41420_2025_2540_MOESM3_ESM.jpg]
